# The giant protein titin regulates the length of the striated muscle thick filament

**DOI:** 10.1038/s41467-017-01144-9

**Published:** 2017-10-19

**Authors:** Paola Tonino, Balazs Kiss, Josh Strom, Mei Methawasin, John E. Smith, Justin Kolb, Siegfried Labeit, Henk Granzier

**Affiliations:** 1 Department of Cellular and Molecular Medicine, University of Arizona, Tucson, Arizona 85721 USA; 2 Sarver Molecular Cardiovascular Research Program, University of Arizona, Tucson, Arizona 85721 USA; 3 Department of Integrative Pathophysiology, Medical Faculty Mannheim, Mannheim, 68167 Germany; 4 DZHK, Mannheim-Heidelberg, 68167 Germany

## Abstract

The contractile machinery of heart and skeletal muscles has as an essential component the thick filament, comprised of the molecular motor myosin. The thick filament is of a precisely controlled length, defining thereby the force level that muscles generate and how this force varies with muscle length. It has been speculated that the mechanism by which thick filament length is controlled involves the giant protein titin, but no conclusive support for this hypothesis exists. Here we show that in a mouse model in which we deleted two of titin’s C-zone super-repeats, thick filament length is reduced in cardiac and skeletal muscles. In addition, functional studies reveal reduced force generation and a dilated cardiomyopathy (DCM) phenotype. Thus, regulation of thick filament length depends on titin and is critical for maintaining muscle health.

## Abstract (web summary)

Thick filaments in skeletal muscle and heart are composed of myosin. The authors show that the length of thick filaments is defined by titin, and that alterations in titin length affect force generation and lead to dilated cardiomyopathy in mice.

## Introduction

The contractile machinery that powers striated muscle (heart and skeletal muscles) has as its most crucial component the thick filament, comprised of the molecular motor myosin^1, 2^. The thick filament is of a precisely controlled length^3^, defining thereby the force level that muscles generate and how this force varies with muscle length^4^. The mechanisms by which the thick filament length is so exquisitely controlled are unclear, and it has been speculated that the giant protein titin could be involved and function as a molecular ‘ruler’^5–8^.

Titin, the largest protein known, spans the half-sarcomere (contractile unit of muscle), from Z-disk to M-band^9^, is modular in structure, and contains ~300 immunoglobulin (Ig)- and fibronectin (Fn)-like domains. The I-band segment of titin contains only Ig domains and several unique sequences^10^, all of which contribute to titin’s elasticity that allows it to function as a complex molecular spring that contributes greatly to the diastolic stiffness of the heart^11^. This spring can be tuned with as prominent tuning mechanism post-transcriptional regulation that results in isoforms with distinct spring region composition^12, 13^. The adult heart coexpresses the small and relatively stiff N2B titin isoform and the longer and more complaint N2BA titin isoform^14^.

**Fig. 1:**
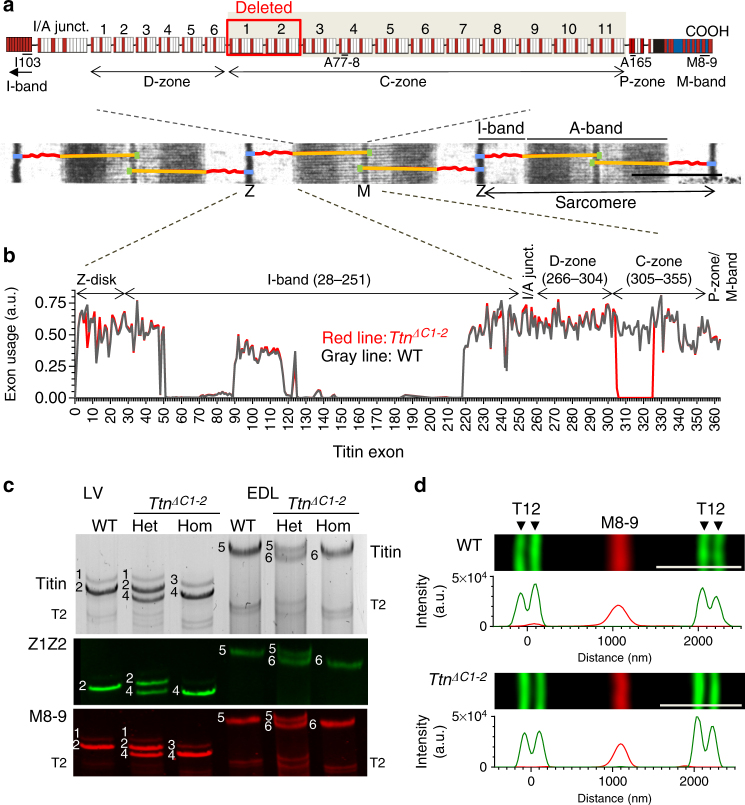
Genetically engineered mouse model lacking two C-zone repeats in titin. **a** Titin spans from Z-disk (Z) to M-band (M) in the sarcomere. Top, domain structure of A-band segment of titin highlighting the C-zone and the two deleted C-repeats in the *Ttn* ^*ΔC1-2*^ mouse model (additionally, showing binding sites of the titin antibodies used in this study). **b** Titin exon usage in myocardial tissue from 8-week-old WT and *Ttn* ^*ΔC1-2*^ male mice (*n* = 8 each). **c** Top, agarose protein gel of titin in left ventricular (LV) myocardium and extensor digitorum longus (EDL) skeletal muscle. Bottom, western blots using Z1Z2 and M8-9 antibodies. 1–4 Cardiac titin (1 N2BA (WT), 2 N2B (WT), 3 N2BA (*Ttn* ^*ΔC1-2*^), and 4 N2B (*Ttn* ^*ΔC1-2*^)); 5–6 EDL muscle titin (5 N2A (WT) and 6 N2A (*Ttn* ^*ΔC1-2*^)). **d** Superresolution microscopy using T12 and M8-9 antibodies. Scale bars, 1 μm

Compared to titin’s I-band region, its A-band segment is not well understood, yet recent landmark sequencing studies in large patient cohorts show that these zones are crucial as countless mutations linked to cardiac and skeletal muscle diseases are found here^12, 15–18^. Titin’s A-band segment is orders of magnitude less extensible than the I-band region of the molecule^19^ and it is unlikely therefore that the A-band segment of titin functions as a molecular spring. Titin’s A-band segment largely consists of Ig and Fn domains that form a 7-domain fixed pattern in the D-zone and an 11-domain fixed pattern in the C-zone (see Fig. 1a with domain organization based on ref. ^10^). The C-zone is most prominent and contains 11 super-repeats of the Ig–Fn–Fn–Ig–Fn–Fn–Fn–Ig–Fn–Fn–Fn pattern. Each super-repeat spans ~43 nm in length^20^, binds to myosin^21^ and myosin-binding protein-C (MyBP-C)^22^, and is referred to as a C-zone repeat^10^. Titin molecules run along the thick filament and each of its super-repeats spans ~43 nm in length, a distance that coincides with the ~43 nm myosin helical repeat^20^. Hence, a popular but untested theory is that in vertebrate animals titin functions as a thick filament template that is responsible for determining thick filament length. A recent study in which a large part of titin near the edge of the A-band was deleted was negative in that the thick filament length was unaltered^19, 23, 24^.

Here we report studies on a mouse model, *Ttn* ^*ΔC1-2*^, in which two of titin’s super-repeats (C1 and C2) were deleted. Structural studies in both cardiac and skeletal muscles of *Ttn* ^*ΔC1-2*^ mice reveal a reduced thick filament length, in line with the concept of a 2 × 43 nm shortened titin ruler. Skeletal muscles of *Ttn* ^*ΔC1-2*^ mice generate less force and have a steeper descending limb of their force–sarcomere length relation, supporting the structural finding of shorter thick filaments. The heart generates less pressure and, unexpectedly, has a dilated cardiomyopathy (DCM) phenotype, a heart disorder characterized by ventricular dilation and depressed contractility^25^ and a common cause of heart failure in humans with a prevalence of up to 1:250^26^. Importantly, there are many truncation mutations in the A-band segment of titin (including 12 within the C1 and C2 repeats) associated with DCM^15, 27, 28^ and these truncation mutations may impact titin’s role in thick filament length regulation, causing a force reduction and leading to DCM. Thus, our work shows for the first time that thick filament length regulation is titin based and is essential for maintaining muscle health.

## Results

### The *Ttn*^*ΔC1-2*^ mouse model

To test the role of titin in thick filament length regulation, homologous recombination was used to delete from the mouse titin gene exons 305–325 (details in Supplementary Fig. 1A). This deletion keeps the reading frame intact but internally deletes from titin 2177 amino acids (239.5 kDa) that code for titin’s C-zone super-repeats 1 and 2. The homozygous *Ttn* ^*ΔC1-2*^ mice are viable and are obtained from heterozygous parents at a Mendelian ratio (Supplementary Fig. 1B). *Ttn* ^*ΔC1-2*^ mice have normal growth curves and muscle weights (Supplementary Fig. 1C, D). An RNA sequencing (RNAseq) analysis revealed that titin exon usage in the adult myocardium is unaltered, except for the absence of the deleted exons 305–325, indicating that no adaptations in splicing occur (Fig. 1b). Protein analysis on both left ventricular (LV) myocardium and extensor digitorum longus (EDL) skeletal muscle revealed that homozygous mice express titin of increased mobility (Fig. 1c), but at normal levels (Supplementary Fig. 2A, B), with also normal myosin expression (Supplementary Fig. 2B–D). An exception is the small but significant upregulation of N2BA titin at the expense of N2B titin in the left ventricle of *Ttn* ^*ΔC1-2*^ mice. Western blots using antibodies to titin’s N terminus (Z1Z2) and C terminus (M8-9) show that these regions are normally expressed in mutant titin (Fig. 1c). Additionally, superresolution optical microscopy (structured illumination microscopy (SIM)) reveals that titin’s N and C termini are normally incorporated in the sarcomere of *Ttn* ^*ΔC1-2*^ mice (Fig. 1d). Thus, except for the internal deletion of two super-repeats, the titin molecule in *Ttn* ^*ΔC1-2*^ mice is intact. The *Ttn* ^*ΔC1-2*^ model that we created is ideal for studying the role that titin plays in thick filament length regulation.

### Thick filament length

Structural studies were focused on left ventricular cardiac muscle and EDL skeletal muscle of 8-week-old homozygous *Ttn* ^*ΔC1-2*^ and littermate wild-type (WT) control mice. In initial studies, the thick filament length was determined from the width of the A-band as measured by electron microscopy (EM). Studies on cardiac muscles stretched by different degrees revealed that the A-band width was significantly reduced in *Ttn* ^*ΔC1-2*^ mice (Supplementary Fig. 3A), with similar findings in EDL muscle (Supplementary Fig. 3B). Immunoelectron microscopy (IEM) was performed next, using the I103 titin antibody that is known in WT muscle to mark titin at the end of the thick filament^19^ near the junction between the A-band and the I-band (location of its binding site in titin is shown in Fig. 1a, see also ref. ^19^). If titin does not regulate thick filament length, then deleting C-zones 1 and 2 is expected to move the I103 epitope inside the A-band by ~86 nm (twice the length of the C-zone repeats). However, in *Ttn* ^*ΔC1-2*^ mice the I103 epitope maintained its location near the edge of the A-band in both cardiac and skeletal muscle (compare left and middle panels of Supplementary Fig. 3C, D) and the graph of epitope-to-epitope distances versus sarcomere length was shifted to shorter distances in the *Ttn* ^*ΔC1-2*^ mice, similar to the shift in A-band width (right panels of Supplementary Fig. 3C, D). Thus, deleting two C-zone repeats shortens both titin and the thick filament by similar amounts.

**Fig. 2:**
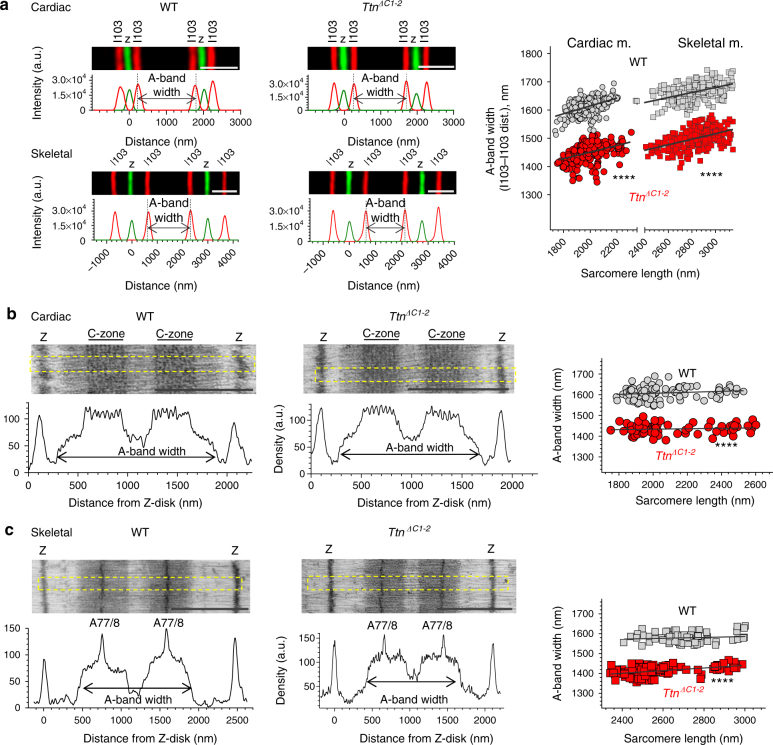
Effects of deleting C-zone repeats on A-band width. **a** Left, example of SIM images obtained using cardiac and skeletal muscle sections labeled with I103 titin antibody (A-band, red) and α-actinin (Z-disk, green). Right, summarized results of the A-band width measurements. In both muscle types the offsets of the linear regression lines are significantly lower in *Ttn* ^*ΔC1-2*^ mice. **b**, **c** Left, representative IEM images of sarcomeres labeled with cMyBP-C (**b**) or A77/8 (**c**) with density profiles shown at bottom. Obtained summarized results are shown to the right. **c** The distance between A77/8 epitopes was measured and used to determine A-band shrinkage in skeletal muscle. Right, Shrinkage-corrected A-band width results. All linear regression fits show positive slopes but none are significantly different from zero. Line offsets are significantly reduced in *Ttn* ^*ΔC1-2*^ mice. Statistical analysis is provided in Supplementary Table 4 (**** denotes *p* < 0.0001). Scale bars, 1 μm. The 8-week-old male mice were used (SIM: 10 WT and 9 *Ttn* ^*ΔC1-2*^, IEM: 4 WT and 4 *Ttn* ^*ΔC1-2*^)

Preparing muscle tissue for transmission electron microscopy (TEM) is known to cause shrinkage during sample preparation^3^; to address whether the reduced thick filament length in *Ttn* ^*ΔC1-2*^ mice might be due to differential shrinkage, two distinct methods were used. The first was based on SIM, a method that unlike EM does not require dehydration and embedding and shrinkage is expected to be different from that in EM studies. For these studies, the I103 antibody was used as it marks the edge of the A-band in both WT and *Ttn* ^*ΔC1-2*^ mice (see above). Epitope-to-epitope distances were found to be reduced in both muscle types of *Ttn* ^*ΔC1-2*^ mice (Fig. 2a), by on average 164 nm in cardiac muscle and 163 nm in skeletal muscle, or ~41 nm per deleted C-zone repeat. Additionally, we performed IEM with a cardiac MyBP-C (cMyBP-C) antibody that labels multiple stripes in the A-band that in vivo are known to be 43 nm apart^29^. By measuring the stripe distance on electron micrographs we determined the degree of tissue shrinkage and used this to correct the measured A-band width values. Large shrinkage values were found in sarcomeres that are at short sarcomere length (Supplementary Fig. 4), consistent with short A-band findings of others^30^. Using cMyBP-C as an ‘internal caliper’ reveals that thick filament length was reduced in *Ttn* ^*ΔC1-2*^ myocardium (Fig. 2b), by 173 nm or ~43 nm per deleted C-zone repeat (Table 1).

**Table 1:** Summary of the A-band width measurements based on IEM

	Cardiac muscle			Skeletal muscle		
	WT	*Ttn* ^*ΔC1-2*^	*p*-value	WT	*Ttn* ^*ΔC1-2*^	*p*-value
SL range (μμ)	~ 1.8–2.5	~ 1.8–2.6		~ 2.4–3.0	~ 2.4–3.0	
A-band width (nm)	1608 ± 30	1435 ± 25	<0.0001	1580 ± 24	1412 ± 24	<0.0001
Δ A-band width (nm)		173 ± 4			168 ± 10	
Δ per C-zone repeat (nm)		43.4			42.0	

Shrinkage-corrected A-band width is significantly shorter by 173 nm in cardiac and 168 nm in skeletal muscle of *Ttn* ^*ΔC1-2*^ mice compared to wild type. Values are mean±s.d. Statistical analysis performed with a two-tailed *t*-test. See Supplementary Table 4 for additional statistical details. A total of 6 WT and 6 *Ttn* ^*ΔC1-2*^ male mice at 8 weeks of age were used

The available skeletal muscle-specific anti MyBP-C antibodies did not provide adequate labeling in EDL muscle, and therefore an alternative shrinkage correction method was developed. An antibody was raised against the first two Fn domains of C-zone 4 (A77-8, Fig. 1a); the real distance between the two epitopes across the M-band can be estimated at 892 nm (for details, see the legend of Supplementary Fig. 5C) and the deviation from the predicted value on electron micrographs was used to determine A-band shrinkage (for details, see Supplementary Fig. 5). Using A77-8 as the ‘internal caliper’ resulted in a thick filament length that was reduced in EDL muscle of *Ttn* ^*ΔC1-2*^ mice (Fig. 2c, right) by 168 nm or 42 nm per deleted C-zone repeat (Table 1). Thus, a range of techniques clearly reveal that deleting two super-repeats from the titin gene results in shorter thick filaments, and the shrinkage-corrected values of ~41–43 nm per half thick filament support the hypothesis that each titin super-repeat specifies 43 nm length increments of the thick filament.

### Functional studies

**Fig. 3:**
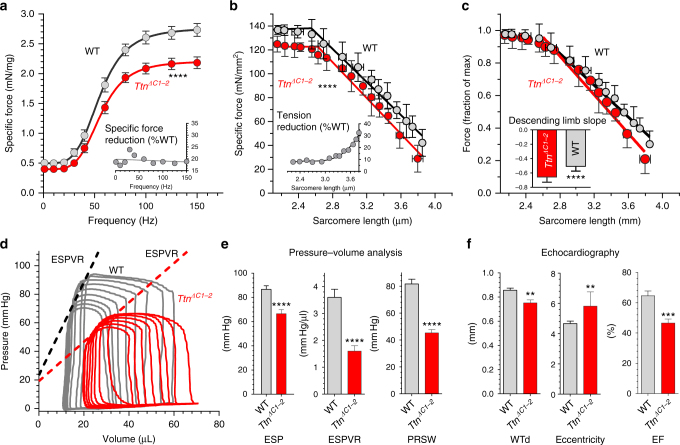
Functional effects of deleting C-zone repeats of skeletal muscle and the heart. **a** In vivo force generation by the gastrocnemius muscle complex. The specific force frequency relation revealed significantly reduced force levels in *Ttn* ^*ΔC1-2*^ mice (see inset). **b**, **c** Force–sarcomere length relations of skinned fiber bundles from EDL muscle activated by maximal calcium levels. **b** Specific force is significantly reduced in *Ttn* ^*ΔC1-2*^ mice (red symbols) with a reduction that, relative to WT levels, increases with sarcomere length (inset). **c** Force normalized to maximal force at optimal length reveals a descending limb with a slope that is significantly steeper in *Ttn* ^*ΔC1-2*^ mice (inset). Mice at 60 days of age were used (*n* = 6 male and 2 female WT; *n* = 7 male and 2 female *Ttn* ^*ΔC1-2*^). **d**, **e** Pressure–volume (PV) analysis of the left ventricular chamber. Sample PV loops in Fig. 3d reveal reduced end-systolic pressures and a reduced slope of the end-systole PV relation (ESPVR) in *Ttn* ^*ΔC1-2*^ mice (red), with summarized results in Fig. 3e. Male mice at 62–74 days of age were used (*n* = 7 WT and *n* = 8 *Ttn* ^*ΔC1-2*^). **f** Echocardiography revealed wall thinning (diastolic wall thickness (WTd)), an increased eccentricity index, and a reduced ejection fraction (EF), all indicating that *Ttn* ^*ΔC1-2*^ mice have dilated cardiomyopathy (DCM). The 50-day-old male mice were studied (*n* = 8 WT and *n* = 8 *Ttn* ^*ΔC1-2*^). Mean values±s.e.m. are shown. Additional statistical analysis is provided in Supplementary Table 4 (** denotes *p* < 0.01; ***p < 0.001, and ****p < 0.0001)

The reduced thick filament length is expected to lower maximal calcium-induced force by 12% (taking the length reduction at 170 nm and the crossbridge bearing part of the thick filament at 1440 nm^4^). Furthermore, the descending limb of the force–sarcomere length relation (with force normalized to the maximal force at optimal sarcomere length) is expected to be steeper due to the reduced length of the crossbridge bearing region of the thick filaments that reduces the distance for thin filaments to slide from 100% to 0% overlap. To test these predicted effects on force, we first carried out an in vivo functional study on skeletal muscle in which the gastrocnemius muscle was stimulated at a range of frequencies (1 to 150 Hz). This revealed a significantly reduced force level in *Ttn* ^*ΔC1-2*^ mice (Fig. 3a), on average 19.4 ± 1.4% (Fig. 3a, inset). Studies were also conducted on demembranated fibers from the EDL muscle that were stretched to a predetermined sarcomere length and then activated with a maximal activating level of calcium (0.1 mM free Ca^2+^). This revealed that calcium-activated force at all sarcomere lengths on the plateau of the force–sarcomere length relation is reduced in *Ttn* ^*ΔC1-2*^ fibers by on average 9.1 ± 0.9% (Fig. 3b). The descending limb of the force–sarcomere length is down-shifted and when forces are expressed relative to WT levels, the force deficit progressively increases with sarcomere length (Fig. 3b, inset), consistent with the descending limb of the normalized force–sarcomere length relation that is steeper (Fig. 3c). These findings support the reduced thick filament length measured in skeletal muscles of *Ttn* ^*ΔC1-2*^ mice.

To evaluate cardiac function, a pressure–volume analysis was conducted by inserting a small 1.2 F conductance catheter into the LV of the heart and recording pressure–volume (PV) loops at a range of end-diastolic volumes (see Methods for details). Sample loops are shown in Fig. 3d and summarized results in Fig. 3e and Table 2. The end-systole pressure (ESP) is reduced in the *Ttn* ^*ΔC1-2*^ mice as is the slope of end-systolic pressure–volume relation (ESPVR) and the preload recruitable stroke work (PRSW), both indexes of myocardial contractility^31^ (Fig. 3e, middle and right). PV studies also revealed that the LV operates at larger volumes in *Ttn* ^*ΔC1-2*^ mice (Table 2). An echocardiography study showed in *Ttn* ^*ΔC1-2*^ mice LV wall thinning, an increased eccentricity index (Fig. 3f, left and middle, Supplementary Table 1), and a reduced ejection fraction (Fig. 3f, right). These findings indicate that *Ttn* ^*ΔC1-2*^ mice have DCM, a disorder characterized by LV chamber dilation and contractile dysfunction^25, 32^.

**Table 2:** Pressure (P)–volume (V) analysis of the left ventricular chamber of the heart

	WT	*Ttn* ^*ΔC1-2*^	*p*-value
*n*	7	8	
HR (b.p.m.)	497 ± 6	463 ± 6	0.0015
ESP (mm Hg)	86.8 ± 2.8	66.7 ± 3.2	0.0004
EDP (mm Hg)	2.5 ± 0.5	2.6 ± 0.7	0.955
dP/dt max (mm Hg/s)	8460 ± 302	5591 ± 200	<0.0001
dP/dt min (mm Hg/s)	−8319 ± 357	−3842 ± 227	<0.0001
ESV (μL)	19.4 ± 3.1	43.6 ± 5.3	0.002
EDV (μL)	59.5 ± 4.6	84.4 ± 5.0	0.003
SV (μL)	40.1 ± 3.9	40.8 ± 2.6	0.88
CO (mL/min)	19.9 ± 1.8	18.9 ± 1.3	0.656
EF (%)	67.9 ± 4.2	49.2 ± 4.1	0.007
EA (mm Hg/μL)	2.23 ± 0.17	1.72 ± 0.17	0.057
Tau Glantz (ms)	9.4 ± 0.5	22.7 ± 2.4	0.0002
PRSW	81.7 ± 3.5	45.3 ± 2.4	*p*<0.0001
ESPVR (ES)	3.6 ± 0.3	1.6 ± 0.2	*p*<0.0001
ESPVR (V0)	−4.9 ± 1.6	−10.9 ± 4.1	0.667
EDPVR (β)	0.031 ± 0.007	0.037 ± 0.008	0.584
Arterioventr. coupling (EA/ES)	0.6 ± 0.1	1.2 ± 0.2	0.039

HR: heart rate; ESP: end-systolic pressure; EDP: end-diastolic pressure; dP/dt max: maximal rate of pressure development; dP/dt min: maximal rate of pressure reduction; ESV: end-systolic volume; EDV: end-diastolic volume; SV: stroke volume; EF: ejection fraction; EA, effective arterial elastance; Tau Glantz: left ventricular relaxation time constant; PRSW: preload recruitable stroke work; ESPVR (ES): slope of end-systolic pressure–volume relation; ESPVR (V0): volume intercept of end-systolic pressure–volume relation; EDPVR (β): exponent of exponential fit of end-diastolic pressure–volume relation.

Male mice at 62–74 days of age were used (*n* = 7 WT and *n* = 8 *Ttn* ^*ΔC1-2*^). Mean values±s.e.m. are shown. Statistical analysis with a two-tailed *t*-test. Additional statistical analysis is provided in Supplementary Table 4

To determine whether depressed contractility might be explained by changes in calcium handling in the *Ttn* ^*ΔC1-2*^ mice, calcium transients were measured in isolated single cardiac myocytes that were twitch activated. No differences were found in the base level of calcium, the calcium amplitude, or in the kinetics of calcium release or uptake (Supplementary Fig. 6 and Supplementary Table 2), supporting that the DCM phenotype is primarily myofilament based.

**Fig. 4:**
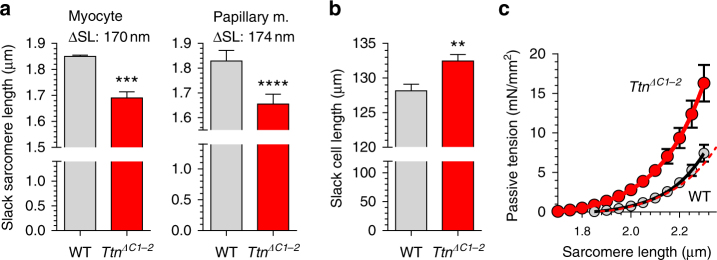
Effect of reduced thick filament length on slack sarcomere length (**a**), slack cell length (**b**), and passive tension (**c**). Skinned myocytes (**a**, left) and skinned papillary muscle (**a**, right) isolated from the left ventricle have shorter slack sarcomere length in *Ttn* ^*ΔC1-2*^ mice, but cell length is longer (**b**). **c** Passive tension of skinned cardiac myocytes as a function of sarcomere length increases steeper in *Ttn* ^*ΔC1-2*^ mice. Individual data points were fit with a fourth-order polynomial function. Dashed red line represents the fit of the *Ttn* ^*ΔC1-2*^ data right-shifted by 173 nm. Note that the right-shifted *Ttn* ^*ΔC1-2*^ fit overlaps well with WT data. **a**, left: mean results from 5 hearts (20 cells per heart); **a**, right: mean results from 12 muscles from 12 mice; **c** mean results from 5 myocytes from 3 hearts. Female mice at 48–56 days of age were used (*n* = 6 WT and *n* = 6 *Ttn* ^*ΔC1-2*^). Error bars are s.e.m. Statistical analysis with a two-tailed *t*-test. Additional statistical analysis is provided in Supplementary Table 4 (** denotes *p* < 0.01, ***p < 0.001, and ****p < 0.0001)

The slack length of sarcomeres and cardiac myocytes was also measured (cells are slack when they are relaxed and do not experience external forces). The slack sarcomere length in *Ttn* ^*ΔC-2*^ myocytes was reduced by 170 nm (Fig. 4a, left), findings that are supported by the 174 nm reduced slack sarcomere length in papillary muscles (Fig. 4a, right). The shorter slack sarcomere length is in contrast to the longer slack length of the myocyte (Fig. 4b), indicating the number of serially linked sarcomeres is increased in *Ttn* ^*ΔC1-2*^ myocytes, based on the measured mean cell length and sarcomere length by 13%. The reduced slack sarcomere length of *Ttn* ^*ΔC1-2*^ myocytes is also expected to give rise to higher passive tensions when passive cells are stretched to a given sarcomere length, consistent with the measured passive tension values (Fig. 4c). The passive tension curves of *Ttn* ^*ΔC1-2*^ and WT myocytes are similar in shape but shifted along the sarcomere length axis. Right shifting the *Ttn* ^*ΔC1-2*^ curve by 173 nm (the difference in A-band width) reproduces the WT curve (see the dashed red curve in Fig. 4c).

## Discussion

The thick filament of vertebrate muscle is of a precisely controlled length, and in the present work we addressed the mechanism by which thick filament length regulation is achieved. Ever since the discovery that titin runs along the thick filament length^9^, and that it contains super-repeats that span a distance that coincides with the ~43 nm myosin helical repeat of the thick filament^20^, it has been speculated that titin functions as a thick filament length ruler^5–8^. To test the role of titin in thick filament length regulation we created the *Ttn* ^*ΔC1-2*^mouse in which two of titin’s super-repeats (C1 and C2) were deleted. Structural studies in both cardiac and skeletal muscles reveal that deleting two super-repeats from the A-band segment of titin reduces thick filament length with each of titin’s C-zone repeats being responsible for a quantal 43 nm thick filament length. Below we discuss the structural and functional findings of this work in detail.

To test the titin ruler hypothesis a mouse model was created in which two of titin’s super-repeats were genetically deleted. Although differently sized deletions could have been made, the titin gene structure limited the available choices as we considered it important to delete an integral number of super-repeats, required introns of sufficiently large size in order to place selection markers, and we had to avoid disrupting the reading frame. Hence, a deletion of two super-repeats was one of our best options. Indeed, an internally shortened mutant titin protein is made by the *Ttn* ^*ΔC1-2*^ mouse that is normally incorporated in the sarcomere (Fig. 1c, d). The deletion is compatible with life, as revealed by breeding heterozygous *Ttn* ^*ΔC1-2*^ parents that produce both *Het* and *Hom* mice at Mendelian ratios and with normal body and muscle weights (Supplementary Fig. 1b–d). A concern when targeting the *TTN* gene is inadvertently causing alterations in splicing of the remaining exons, but the RNAseq-based exon map did not reveal changes in exon usage in LV myocardium (Fig. 1b), indicating that no major changes in splicing had occurred. However, at the protein level small but significant differences in isoform expression were found: the N2BA titin isoform was upregulated relative to the N2B isoform (Supplementary Fig. 2A). This combined with the absence of alterations in titin at the transcript level suggests changes in protein turnover, i.e., a slowdown in degradation of the N2BA isoform and an increase in degradation of the N2B isoform. These two isoforms have the same exon composition in the A-band and Z-disk regions of the molecule but differ in their I-band regions^33, 34^. The I-band region of the N2BA titin molecule has, compared to N2B titin, additional exons spliced in, and is therefore more compliant (i.e., it generates less force for a given extension)^35, 36^. The upregulation of N2BA titin that was found in the *Ttn* ^*ΔC1-2*^mouse can be viewed as a beneficial adaptation that offsets the increased passive stiffness that is expected from the shortened A-band in the *Ttn* ^*ΔC1-2*^mouse (see below).

Determining the thick filament length from TEM images requires a correction for the shrinkage that is well known to take place during sample preparation^3^. Large levels of A-band shrinkage were found in our study in both cardiac and skeletal muscles, with a degree of shrinkage that was ~20–30% in short sarcomeres (Supplementary Figs. 4 and 5). As sarcomere length was increased, the degree of shrinkage was reduced (in sarcomere >~2.5 μm to 5–10% shrinkage), explaining the large slopes of the regression lines fit to the A-band width versus sarcomere length data (Supplementary Fig. 3). Correcting for shrinkage largely abolishes this sarcomere length dependence and results in a mean thick filament length in WT mice of 1.61 μm in cardiac and 1.58 μm in skeletal muscle (Table 1). These values are in line with the 1.62 μm reported by Huxley and colleagues^3^, using a specialized rapid freeze/freeze substitution technique, indicating that the shrinkage correction methods used in our work are valid. The explanation for the finding that the level of shrinkage is reduced as sarcomere length is increased and that shrinkage is less in the *Ttn* ^*ΔC1-2*^ model (Supplementary Figs. 4B and 5D) is unclear. We speculate that the titin-based force that pulls outward on the thick filaments counters shrinkage, as this force increases with sarcomere length and is higher in *Ttn* ^*ΔC1-2*^ mice (Fig. 4c). It is also worthwhile noting that the cMyBP-C antibody that was used to study shrinkage revealed in myocardium of WT mice 9 cMyBP-C stripes (consistent with findings by others^29, 37^) but in *Ttn* ^*ΔC1-2*^ mice the outer stripe (stripe 9) was absent (with occasional evidence for spotty labeling). This suggests that titin super-repeat 1 must be different from the others, and is not involved in anchoring cMyBP-C, but that super-repeat 2 is. This is unexpected since many of the thick filament models locate cMyBP-C on titin super-repeats 1–9^29^, warranting follow-up studies on the cMyBP-C-binding properties of the terminal titin super-repeats.

Importantly, following shrinkage correction the thick filament length is reduced in *Ttn* ^*ΔC1-2*^ mice by 173 nm in cardiac muscle and 168 nm in skeletal muscle (Table 1). Considering that each half thick filament is associated with a titin molecule, the thick filament length reductions that we obtained are in line with the concept of a 2 × 43 nm shortened titin ruler. Thus, for the first time highly supportive evidence exists for titin as a molecular ruler. To explain the underlying mechanism, at least two possibilities present themselves. First, a sequence N terminal to the deleted C-zones might determine the thick filament length and its distance to the M-band is set by the combined length of the C-zone repeats^8^. Because eliminating the I/A junction of titin does not alter thick filament length^19^, its role is unlikely, but a role for the D-zone of titin cannot be ruled out as of yet. Second, the C-zone repeats might play a more direct role in thick filament length regulation, as suggested by the interactions between the Fn domains of the C-zone super-repeats and myosin^21^ and consistent with the recently proposed hypothesis that titin stabilizes the thick filament^7^. Considering that it is likely that there are 6 titin molecules that run along the thick filament^38^, spread around its periphery^1^, we speculate that this interaction between C-zone repeats and myosin holds the thick filament in a ‘cage’ (akin to a Chinese finger trap) that stabilizes the thick filament and prevents depolymerization.

A shortened thick filament is predicted to have multiple functional effects, including a reduction in the slack sarcomere length. The slack sarcomere length is determined by the end-to-end length of titin’s I-band region at which titin exerts no force, a length that is expected to be the same in WT and *Ttn* ^*ΔC1-2*^ myocytes, plus the length of the thick filament. Thus, the shortened thick filament in *Ttn* ^*ΔC1-2*^ myocytes/muscles is predicted to lower the slack sarcomere length by ~170 nm, which is consistent with the measured values (Fig. 4a). The reduced slack sarcomere length in the *Ttn* ^*ΔC1-2*^ myocytes is expected to increase the number of serially linked titin molecules per unit muscle length by ~9% (reduction in slack sarcomere length (170 nm) divided by the slack sarcomere length (~ 1850 nm)). This increased number of titin molecules is likely to explain why the reduced size of titin in the *Ttn* ^*ΔC1-2*^ mouse does not result in the expected ~8% reduction in the total level of titin protein (the deletion of the two super-repeats, 239.5 kDa, divided by the Mw of WT N2B titin, ~3000 kDa) (Supplementary Fig. 2B). Thus, despite the titin molecule is smaller, the total titin protein level will not be reduced, because the number of serially linked titin molecules per unit length of muscle is increased. A similar argument can be made for myosin, the expression level of which is expected to be reduced by ~11% in the *Ttn* ^*ΔC1-2*^ mouse (length reduction of thick filament (~170 nm) divided by thick filament length (~1600 nm)) yet the measured expression level shows no differences (Supplementary Fig. 2B), i.e., the reduced slack sarcomere length increases the number of thick filaments per unit muscle length. As expected, the shorter slack sarcomere length also increases the level of passive tension in stretched cardiac myocytes of *Ttn* ^*ΔC1-2*^ mice, relative to that of WT mice (Fig. 4c). However, what is unexpected is that this is not reflected in the diastolic chamber stiffness (the β−coefficient of the EDPVR is unaltered, Table 2). This contrasts previous work in which titin was genetically altered to either increase titin-based tension^19, 39, 40^ or lower titin-based tension^41, 42^ and LV diastolic chamber stiffness was found to be altered accordingly, findings that have led to the view that titin is a dominant contributor to diastolic chamber stiffness. A possible explanation for the discordance between the results of cardiac myocytes and that of the LV chamber in the present work is a sarcomere length operating range that is shifted to shorter sarcomere lengths in *Ttn* ^*ΔC1-2*^ mice compared to WT mice, i.e., a shift of ~170 nm would result in an identical titin-based passive tension of the myocytes and thus an unaltered chamber stiffness. In summary, shortening the thick filaments reduces the slack sarcomere length and increases the passive tension in stretched myocytes, but this does not cause an increase in diastolic chamber stiffness in *Ttn* ^*ΔC1-2*^ mice, likely because the sarcomere length range during the cardiac cycle is shifted to shorter sarcomere lengths.

Active tension measurements revealed that skeletal muscles of *Ttn* ^*ΔC1-2*^ mice generate less force and have a steeper descending limb of their force–sarcomere length relation. Additionally, the heart generates less systolic pressure and has reductions in the slope of the ESPVR and the PRSW, both indexes of myocardial contractility^31^. Measurements in cardiac myocytes show that calcium transients are unaltered indicating that the depressed contractility of *Ttn* ^*ΔC1-2*^ mice is not due to a reduction in activating calcium levels. Instead, the depressed contractility is consistent with the shorter thick filaments that our structural studies revealed, as fewer force generating myosin molecules in the shortened thick filament will limit the active force level that can be attained. Thus, the depressed contractility of skeletal and cardiac muscles in *Ttn* ^*ΔC1-2*^ mice shows that thick filament length regulation is functionally important for optimal force generation.

Compared to WT mice, the LV chamber of *Ttn* ^*ΔC1-2*^ mice has a dilated phenotype, as revealed by the increased diastolic LV chamber dimension, the decreased LV wall thickness, and the increased eccentricity index (Supplementary Table 1), and supported by the elongation of the cardiac myocytes of *Ttn* ^*ΔC1-2*^ mice (Fig. 4c). In the recent study by Davis et al.^43^, a mathematical model of hypertrophic versus dilated cardiomyopathy was proposed in which the level of myofilament tension is critical in the growth decision that myocytes make with a reduction in tension resulting in eccentric growth and a dilated phenotype. Our finding of a dilated phenotype in the *Ttn* ^*ΔC1-2*^ mouse that has a reduced tension due to shortened thick filaments supports this model. The underlying mechanism is unclear but analogous to what has been reported in other DCM models^43^ that impaired MEK1/ERK signaling might be involved and should be examined in future work, including the possible role of titin’s I-band region that is known to interact with components of this signaling pathway^44^. Finally, several recent landmark next-generation sequencing studies revealed that truncation variants in the titin gene (*TTNtv*) are prevalent in DCM patients^15–18, 27, 45^. Interestingly, most of the *TTNtvs* are found in the A-band segment of titin (including 12 within the C1 and C2 repeats) but how they cause DCM is unknown. We propose that these truncation mutations impact titin’s role in thick filament length regulation and that this causes a force reduction that triggers growth decisions that lead to DCM.

In summary, our studies reveal for the first time the important role of titin in regulating thick filament length, with each of titin’s C-zone repeats being responsible for a quantal 43 nm thick filament length. We conclude that thick filament length regulation is titin based and that this mechanism is crucial for maintaining muscle health.

## Methods

### Generation of the *Ttn*^*ΔC1-2*^ mouse model

All experiments in this study were conducted in accordance with the National Institutes of Health (NIH) Guide for the Care and Use of Laboratory Animals, and all protocols were approved by University of Arizona’s Institutional Animal Care and Use Committee. The *Ttn* ^*ΔC1-2*^ mice were produced via homologous recombination by the Genetically Engineered Mouse Model Core (University of Arizona, Tucson, AZ, USA). The targeting vector was assembled using PCR from C57BL/6J genomic DNA (Jackson Laboratory) into a vector which includes an FRT-flanked neoR selection cassette. After transformation by electroporation and G418 selection of 129S6 ES cells the targeted clones were identified and injected into C57BL/6J blastocysts. Germline transmission was recovered from 1 chimera (verified by PCR). The neoR cassette was removed by mating to ActinB:FLPe mice (Jackson), offsprings were then backcrossed to C57BL/6J. The deleted genomic DNA corresponds to an internal deletion of 2177 amino acids (239.5 kDa).

### Body weight analysis and muscle dissection

Body weight data from WT (18 males, 14 females), *Ttn* ^*ΔC1-2*^ (17 males, 10 females), and *Het* (30 males, 22 females) mice were collected from 16 days to 3 months after birth. Dissection of muscles was performed on heparinized mice (10 U/g, USP, Hospira Inc.) anesthetized with isoflurane (USP, Phoenix Pharmaceuticals, Inc.) and killed by cervical dislocation. The hearts were removed, and both atria and the right ventricle were rapidly dissected and weighed. LV papillary muscles were dissected and skinned in relaxing solution ((in mM): 40 BES, 10 EGTA, 6.56 MgCl_2_, 5.88 Na-ATP, 1 dithiothreitol (DTT), 46.35 K-propionate, 15 creatine phosphate, pH 7.0) containing 1% (w/v) Triton X-100 and protease inhibitors ((in mM): 0.1 E64, 0.47 leupeptin and 0.25 phenylmethylsulfonyl fluoride (PMSF)). The following skeletal muscles were dissected and rapidly weighed: m. tibialis cranialis, m. EDL, m. soleus, m. plantaris, m. gastrocnemius, and m. quadriceps. Both tibias were removed, and the mean tibia length was used for normalization of muscle weight data. Papillary and EDL muscles were skinned overnight at 4 °C in relaxing solution containing 1% Triton X-100 and protease inhibitors, washed thoroughly with relaxing solution, and stored in 50% glycerol/relaxing solution at −20 °C and used within 2 weeks for experiments. All other muscles were quick frozen in liquid nitrogen and stored at −80 °C.

### Titin exon usage

Total RNA was isolated from LV apex of 2-month-old WT and *Ttn* ^*ΔC1-2*^ male (*n* = 6 each) mice using the RNeasy Fibrous Tissue Mini Kit (Qiagen). A Bioanalyzer (Agilent) was used to verify RNA quality and integrity and to determine concentration. Three samples per genotype were prepared with equal amounts of total RNA from two mice per sample. Library preparation and RNAseq were performed by the University of Chicago Genomics Facility following Illumina protocols for RiboZero depletion, TruSeq single-stranded total RNA library construction, and sequencing (2 × 100 bp paired-end reads) on the HiSEQ4000. The sequencing depth was sufficient to yield robust transcript level measurements with a per-sample mean of 40.0±0.5 × 10^6^ paired-end reads aligned to the GRC38v11/mm10 *Mus musculus* reference genome. Analysis was performed on the Illumina BaseSpace cloud platform, the RNAExpress app was run which uses an analysis pipeline with the STAR short read aligner (which maps reads based on the GRC38/mm10 genome including identification of splice sites used)^46^ which outputs counts for splice junction usage; counts for the titin region on chromosome 2 were extracted to produce Fig. 1b.

### Quantification of protein expression

Flash frozen LV and EDL tissues were pulverized in liquid nitrogen and then solubilized in urea buffer ((in mol/L): 8 urea, 2 thiourea, 0.05 tris-HCl, 0.075 dithiothreitol with 3% SDS and 0.03% bromophenol blue, pH 6.8) and 50% glycerol with protease inhibitors ((in mmol/L): 0.04 E64, 0.16 leupeptin and 0.2 PMSF) at 60 °C for 10 min^47^. Samples were centrifuged at 13,000 RPM for 5 min, aliquoted, flash frozen in liquid nitrogen, and stored at −80 °C. Titin isoform analysis was performed on solubilized samples (from *n* = 7 male and 3 female WT; *n* = 7 male and 2 female *Ttn* ^*ΔC1-2*^ 58–75-day-old mice) using a vertical SDS-agarose gel system as previously described^48^. Then, 1% gels were run at 15 mA per gel for 3:20, then stained using Coomassie brilliant blue, scanned using a commercial scanner, and analyzed with One-D scan (Scanalytics Inc.). The integrated optical density (IOD) of titin and major histocompatibility complex (MHC) were determined as a function of loading volume (in a range of 5 volumes). The slope of the linear relationship between IOD and loading was obtained for each protein to quantify expression ratios. For western blotting, solubilized samples were run on a 0.8% agarose gel, then transferred onto polyvinylidene difluoride membranes using a semi-dry transfer unit (Trans-Blot Cell, Bio-Rad). Blots were stained with Ponceau S to visualize the total protein transferred. Blots were then probed with primary antibodies followed by secondary antibodies conjugated with infrared fluorescent dyes. Blots were scanned using an Odyssey Infrared Imaging System (Li-COR Biosciences). The primary antibodies that were used for western blotting, superresolution SIM, and IEM are shown in Supplementary Table 3. Uncropped western blot images using Z1Z2 antibody (Supplementary Fig. 7) and M8-9 antibody (Supplementary Fig. 8) are provided. For MHC protein expression, *n* = 8 WT and *n* = 8 *Ttn* ^*ΔC1-2*^ 58–75-day-old mice (6 males and 2 females in each group) were used.

### Superresolution SIM (SR-SIM)

WT (*n* = 10) and *Ttn* ^*ΔC1-2*^ (*n* = 9) male mice aged 65 days were used in the SR-SIM study. Skinned cardiac and EDL myofibril bundles were embedded in optimal cutting temperature (OCT) compound and immediately frozen in 2-methylbutane precooled in liquid nitrogen. Then, 5 μm thick cryosections were cut and mounted onto microscope slides. Tissue sections were permeabilized in 0.2% Triton X-100/phosphate-buffered saline (PBS) for 20 min at room temperature, blocked with 2% bovine serum albumin (BSA) and 1% normal donkey serum in PBS for 1 h at 4 °C, and incubated overnight at 4 °C with primary antibodies diluted in blocking solution. The primary antibodies included (Supplementary Table 3): a rabbit polyclonal anti-Titin I103 (3.33 μg/mL), a mouse monoclonal anti-Titin T12 (2.5 μg/mL) (Boehringer Mannheim), a mouse monoclonal anti-α-actinin (1:4000) (EA-53, Sigma-Aldrich) antibody, and a guinea pig anti-Titin A165 antiserum (enzyme-linked immunosorbent assay titer: >1:200,000, dilution: 1:50). Sections were then washed with PBS for 2 × 30 min and incubated with secondary antibodies diluted in PBS for 3 h at room temperature. The secondary antibodies (dilution: 1:500), obtained from Invitrogen and Abcam, included: AlexaFluor-488 conjugated goat anti-mouse IgG, AlexaFluor-568 conjugated goat anti-rabbit IgG, AlexaFluor-568 conjugated goat anti-guinea pig IgG, and AlexaFluor-647 conjugated donkey anti-rabbit IgG. The sections were then washed with PBS for 2 × 15 min and covered with number 1.5H coverslips using ProLong Diamond (Thermo Scientific, Inc.). A Zeiss ELYRA S1 SR-SIM microscope was used with ultraviolet and solid-state laser (488/561/642 nm) illumination sources, a 100 × oil immersion objective (NA = 1.46), and a sCMOS camera. Typical imaging was performed on a 49.34 × 49.34 μm^2^ area with 1280 × 1280 pixel dimensions. Image stacks comprising of 30 slices were acquired with 0.101 μm Z-steps, five angles, and five phases/angle for each slice. Image reconstruction and fluorescence intensity plot profile generation were performed with ZEN 2 software (Zeiss). Plot profiles were fit with Gaussian curves to determine the epitope peak position using Fityk 1.3.0 software. A-band width was determined from the I103 epitope positions across the Z-disk. The A165 epitope locations used for shrinkage correction were measured across the A-band.

### TEM and IEM

Skinned LV papillary muscle and EDL skeletal muscle of ~8-week-old male homozygous *Ttn* ^*ΔC1-2*^ mice (*n* = 6) and littermate WT controls (*n* = 6) were stretched from the slack length at different degrees (~20% to 50%) and processed for TEM. Briefly, fixation of muscle tissue was performed with 3% paraformaldehyde in 10 mM PBS, pH 7.2, for 30 min at 4 °C. This was followed by a secondary fixation with 3% glutaraldehyde and 0.02% tannic acid^49^ in the same buffer, and postfixed in 1% OsO_4_ in PBS for 30 min at 4 °C. Samples were then dehydrated in an ethanol graded series, infiltrated with propylene oxide, and transferred to a mix of 1:1 propylene oxide/Araldite 502/Embed 812 (Epon-812, EMS). Subsequently, samples were transferred to a pure Araldite 502/Embed 812 resin and polymerized for 48 h at 60 °C. Ultrathin, 60 nm longitudinal sections were obtained with a Reichert-Jung ultramicrotome and contrasted with 1% potassium permanganate^50^ and lead citrate. Samples were observed in a TECNAI Spirit G2 transmission electron microscope (FEI) and images acquired with a side-mounted AMT Image Capture Engine V6.02 (4Mpix) digital camera operated at 100 kV. Digital images were stored for A-band width measurements with ImageJ 1.49 v (NIH, USA).

Ultrastructural immunolabeling of I103 titin, cMyBP-C (C5–C7), and A77-8 (C-zone 4) epitopes was performed on skinned cardiac and skeletal muscle from WT mice (*n* = 9) and *Ttn* ^*ΔC1-2*^ mice (*n* = 9) stretched to different degrees from the slack length by the preembedding technique with modifications^51^. Fiber bundles from LV muscle and EDL skeletal muscle were skinned twice and washed in relaxing solution as for TEM, previous to fixation with 3% paraformaldehyde in PBS for 30 min at 4 °C, followed by washes with PBS, and with PBS containing protease inhibitors. Blocking was performed with different concentrations of BSA (1%, 0.5%, 0.25%) in PBS containing protease inhibitors and 0.05% Tween-20, according to the used primary antibodies (Supplementary Table 3) from rabbit: anti-I103 (1:25, 24 h), anti-cMyBP-C (1:8, 72 h), and anti-A77-8 (GenScript, 1:20, 48 h), respectively. After rinsing with PBS containing protease inhibitors, muscles were incubated with the secondary antibodies Fab goat anti-rabbit antibody IgG (AP 132, Millipore, 1:25) or AlexaFluor-568 goat anti-rabbit IgG (ab175471, Invitrogen, 1:30) as appropriated. All incubations were performed in a humid chamber at 4 °C. Labeling of cardiac muscle with cMyBP-C was performed on bundle fibers untreated and treated with 1:2 of relaxing solution/gelsolin (a recombinant 45 kDa N-terminal fragment that is calcium independent^52^, 0.6 mg/mL (in mM): 25 MOPS, 150 KCl, 1 DTT, 1 EGTA, pH 7.4) with continuous shaking for 1 h at 4 °C to extract thin filaments, previous to the fixation step. Thin filament extraction was used in the experiments on cardiac muscle to make it easier for the cMyBP-C antibody to reach its binding site and, additionally, to enhance the contrast between A-bands and I-bands, making it easier to measure thick filament length (e.g., Fig. 2b and Supplementary Fig. 4A). Skeletal muscle fibers were hard to extract, confirming previous findings^53^ and were therefore studied with thin filaments in place (e.g. Fig. 2c and Supplementary Fig. 5A). To test whether thin-filament extraction affects the results we also performed experiments on cardiac muscle that were not gelsolin treated. We obtained results that were indistinguishable from fibers that had been gelsolin treated. After labeling, muscle tissues were washed in PBS and fixed with 3 % glutaraldehyde in the same buffer, then processed for TEM and images recorded as explained above. Digital images were calibrated with ImageJ and the density plot profiles were analyzed to determine the width of A-band from the following measurements: I103 titin epitope-to-epitope distance on cardiac and skeletal muscle, the spacing between cMyBP-C-labeled stripes on cardiac muscle, and the A77-8 epitope-to-epitope distance on skeletal muscle across the M-band. These measurements were used to determine the degree of A-band shrinkage to convert measured A-band into shrinkage-corrected A-band width values, and then analyzed with linear regression to test differences from slopes linear fits and line offsets.

### In vivo pressure-volume measurements

An in vivo PV analysis was performed on 62–74-day-old male mice (*n* = 7 WT and *n* = 8 *Ttn* ^*ΔC1-2*^) using an Advantage Admittance Derived Volume Measurement System (SciSense, Inc.) and 1.2 F catheters with 4.5 mm electrode spacing. Mice were anesthetized and ventilated with 3% isoflurane for induction and 1.5% for maintenance with body temperature maintained at 37 °C. (Note that the level of sedation varies slightly among animals and that therefore we will not assign importance to small differences in heart rates between groups.) Anesthetized mice were secured and a bilateral subcostal incision was made. The diaphragm was opened to expose the heart. The catheter was inserted into the LV via apical approach. The inferior vena cava was located and occluded during a sigh (pause) in ventilation to acquire load-independent indexes. Data acquisition and analysis were performed in LabScribe2 (iWorx). EDPVR was analyzed using a monoexponential fit (*P*=C+Ae^βV^) with the exponent (β) reported as the stiffness^31^. Details are in Supplementary Table 1.

### Mouse echocardiography

Male WT (*n* = 8) and *Ttn* ^*ΔC1-2*^ (*n* = 8) mice at 50 days of age were anesthetized with 1% isoflurane, then placed in dorsal recumbence on a heated platform (body temperature: 37 °C). Transthoracic echo images were obtained with a Vevo 2100 High-Resolution Imaging System (Visual-Sonics, Inc.) using the model MS550D scan head for cardiac imaging and MS250 to measure aortic flow velocity. Care was taken to avoid animal contact and excessive pressure which could induce bradycardia. Imaging was performed at a depth setting of 1 cm. Images were collected and stored as a digital cine loop for offline calculations. Standard imaging planes and functional calculations were obtained according to American Society of Echocardiography guidelines. The parasternal long-axis view and mid-wall cross-sectional view of the LV were used to guide calculations of percentage fractional shortening, percentage ejection fraction, and ventricular dimensions and volumes. The left atrial dimension was measured in the long-axis view directly below the aortic valve leaflets. Passive LV filling peak velocity, *E* (cm/s), and atrial contraction flow peak velocity, *A* (cm/s), were acquired from the images of mitral valve Doppler flow from tilted parasternal long-axis views. A sweep speed of 100 mm/s was used for M-mode and Doppler studies. The heart rates of animals during the echocardiographic study were maintained in the range of 500–550 beats/min for M-mode, 450–500 beats/min for B-mode, and 350–450 beats/min for Doppler studies. For details see Supplementary Table 2.

### Cell isolation and calcium transients

The 48–56-day-old female mice (6 WT and 6 *Ttn* ^*ΔC1-2*^) were heparinized and killed by cervical dislocation under isoflurane. The heart was removed and cannulated via the aorta for retrograde coronary perfusion^54^. The heart was perfused for 4 min with perfusion buffer, followed by 20 min digestion using 0.05 mg/mL Liberase TM and 13 µM CaCl_2_ in perfusion buffer. Digestion was halted by placing the heart in myocyte stopping buffer (0.08 mg/mL bovine calf serum and 8 µM CaCl_2_ in perfusion buffer with protease inhibitors). The LV was cut into small pieces, triturated several times with a transfer pipette, and then filtered through a 300 µm nylon mesh filter to acquire intact cardiomyocytes. Ca^2+^ (1 mM final concentration) was reintroduced to the cardiomyocyte suspension. Isolated LV cardiomyocytes were incubated for 10 min at room temperature with 2 µM Fura-2 AM (F-1225, Life Technologies, Inc.) in perfusion buffer containing 0.08 mg/mL bovine calf serum and 1 mM CaCl_2_ and resuspended in 1.8 mM Ca^2+^ Dulbecco’s modified Eagle’s medium (DMEM)/F-12. All intact cell experiments were performed at 37 °C in DMEM/F-12 plus 10 μg/mL insulin in a temperature-controlled flow chamber (flow rate ~2 mL/min) equipped with platinum electrodes mounted on an Olympus IX-70 inverted microscope with a 40× objective. Cells were field-stimulated at 2 Hz frequency. Data were collected with an IonOptix FSI A/D board and IonWizard 6.2.2.61 software with SarcLen modules to determine sarcomere length. Fura-2 fluorescence was measured ratiometrically at 510 nm subsequent to alternate excitations at 340 and 380 nm. Background fluorescence was subtracted for each excitation wavelength. The ratio of fluorescence intensities excited at 340 nm and 380 nm was used as a relative measurement of cytoplasmic Ca^2+^, and the ratio transient was fitted by the IonWizard monotonic transient analysis software.

### In vivo muscle analysis

The function of the gastrocnemius muscle complex was studied in vivo on 57–62-day-old WT (6 male and 2 female) and *Ttn* ^*ΔC1-2*^ (7 male and 2 female) mice using a Mouse Muscle Physiology System (model 809B; Aurora Scientific Inc.). Mice were anesthetized using isoflurane and placed on a heated platform (39 °C). Hair was removed from the right hind-leg and the knee immobilized using a noninvasive clamp. The foot was secured to the footplate on the force transducer with adhesive tape and set at a 90° angle. Needle electrodes were placed just under the skin on either side of the tibial nerve, distal to the knee. Forces were recorded using ASI 610A Dynamic Muscle Control v5.5 software. The Isometric force–frequency relationship was measured by delivering optimal current at 1, 10, 20, 30, 40, 60, 80, 100, 125, and 150 Hz stimulation frequencies. Maximal tetanic force was achieved at 150 Hz. For obtaining the force–frequency relationship, both raw and normalized data were grouped based on genotype and results were fit with a with a four-parameter Hill equation. Tissue weights of the contralateral gastrocnemius complex (gastrocnemius, plantaris, and soleus) were used for force normalization.

### Skinned myocytes mechanics

LV myocytes were isolated as explained above (see ‘Cell Isolation and calcium transients’) and were then skinned. Myocytes were skinned for 7 min in relaxing solution ((in mmol/L) 40 BES, 10 EGTA, 6.56 MgCl_2_, 5.88 Na-ATP, 1.0 DTT, 46.35 K-propionate, 15 creatine phosphate, pH 7.0) with protease inhibitors ((in mmol/L) 0.4 leupeptin, 0.1 E64, and 0.5 PMSF) and 0.3% Triton X-100 (Ultrapure; Thermo Fisher Scientific). Cells were washed extensively with relaxing solution pCa 9 and stored on ice. Myocytes were added to a room temperature flow-through chamber mounted on the stage of an inverted microscope (Diaphot 200; Nikon). Skinned myocyte was glued at one end to a force transducer (Model 406A or 403A, Aurora Scientific). The other end was bent with a pulled glass pipette attached to micromanipulator so that the myocyte axis aligned with the microscope optical axis and cross-sectional area (CSA) was measured directly. The cross-sectional images of skinned cells were analyzed by ImageJ 1.41 software (National Institutes of Health) and were used to convert measured force to stress and for cell dimension study (Supplementary Fig. 4d). Then, the free end of the cell was glued to a servomotor (Model 308B, Aurora Scientific) that imposes controlled stretches. Sarcomere length (SL) was measured with a MyocamS and SarcLen acquisition module (IonWizard 6.2, IonOptix Co., MA, USA) attached to a computer. Passive tension was measured in relaxing solution pCa 9 with protease inhibitors at room temperature. Cells were stretched from their slack length at a speed of 1 base length/s followed by a 20 s hold and then a release back to the original length. The recovery time of at least 7 min in between stretches was utilized to prevent memory effects in subsequent measurements. Data were collected using a custom LabVIEW VI (National Instruments, Austin, TX, USA) at a sample rate of 1 kHz. Measured forces were converted to stress (force/unit undeformed CSA). The stress during the 1 base length/s stretch was plotted against the SL and fitted with a monoexponential curve to derive stress–SL relationships.

### Skinned muscle fiber bundle mechanics

A total of 5 WT and 5 *Ttn* ^*ΔC1-2*^ mice (3 males and 2 females in each group) at 60 days of age were used in the skinned muscle mechanics experiments. Fiber bundles from skinned EDL tissue were dissected for mechanics experiments and mounted using aluminum T clips between a length motor and a force transducer in an 802D Permeabilized Fiber Test Apparatus (Aurora Scientific Inc.) on a Zeiss Axio Observer A1 inverted microscope. SL was set using a high-speed VSL camera controlled by ASI 600A software (Aurora Scientific). Fiber bundles were maintained at 8 °C, and the temperature was temporarily increased to 15 °C during activation. Fibers were placed in relaxing solution (pCa 8.5), then preactivated in relaxing solution with reduced 1 mM EGTA, and activated in pCa 4.5 activating solution ((in mM): 40 BES, 10 CaCO_3_-EGTA, 6.29 MgCl_2_, 6.12 Na-ATP, 1 DTT, 45.3 K-propionate, 15 creatine phosphate, and protease inhibitors) at SL = 2.4 µm to record maximal active tension. The resting slack SL was readjusted to 2.4 μm after each activation–relaxation cycle. Specific force was expressed as force per CSA assuming elliptical fiber cross-section. Most activations had internal SL shortening during force development and in some fiber bundles an initial shortening phase was followed by a slow stretch phase (most likely due to sarcomeres outside the field of view that were stronger and that continued to shorten) that was accompanied by a slow force creep. In those cases force was recorded prior to the onset of the creep phase. To establish the force–SL relationship on the descending limb, fiber bundles were sequentially activated at a range of sarcomere lengths and SL was recorded both prior to and during activation. The passive force–SL curves were determined based on the average of pre- and post-activation passive forces for each SL. Internal shortening correction was applied to passive forces during activation by using nonlinear standard curve interpolation. The passive force during activation was then subtracted from the maximal active force, isolating the active force generating potential for each fiber bundle at a given SL.

### Statistics

For animal studies the sample size was calculated by performing a power analysis using G*Power 3.1.9.2 software (written by Franz Faul, Universität Kiel, Germany). The genotype of the animals included in the study was determined by sequence-specific PCR (see Methods/Generation of the *Ttn* ^*ΔC1-2*^ mouse model) as well as protein gel electrophoresis after the experiment (see Methods/Quantification of protein expression). Sample randomization as well as blinding of the investigator was applied in the case of in vivo animal studies, isolated cell experiments, and skinned muscle mechanics where investigators were unaware of the genotype of the animals during the experiments. Statistical analysis was performed using GraphPad Prism 6 or 7 (GraphPad Software, Inc.). Descriptive statistical results are shown as mean±s.e.m. unless stated otherwise. Differences between groups were considered to be statistically significant at a probability value of *p* < 0.05. A two-tailed *t*-test was used when comparing two groups only, and Welch’s correction was applied in the case of unequal variances between the two groups. One-way analysis of variance using Bonferroni post hoc analysis was performed to assess differences between multiple groups. In order to increase the statistical power of the tests equal or close to equal sample size was applied within independent groups. Linear regression analysis was used to fit and compare EM, IEM, and SR-SIM epitope distance data (Fig. 2). Nonlinear curve fit was applied to the growth curves of animals (Supplementary Fig. 1C) and to the summarized shrinkage data of cardiac (Supplementary Fig. 4B) and skeletal (Supplementary Fig. 5B) muscle. Functional tests required both linear and nonlinear regression analyses to be performed (Fig. 3a–d). The genotype ratio of offspring from *Het*×*Het* breeding was studied by χ^2^ test (Supplementary Fig. 1B). For detailed statistical information see Supplementary Table 4.

### Data availability

All data generated or analyzed during this study are included in this published article (and its supplementary information files) and are available from the corresponding author on reasonable request.

## Electronic supplementary material

Supplementary Information
