# Supplementary material for: The giant protein titin regulates the length of the striated muscle thick filament
Source: Nat Commun. 2017 Oct 19;8:1041. doi: 10.1038/s41467-017-01144-9 (PMC5648799; doi:10.1038/s41467-017-01144-9)
Supplement: Supplementary file 1 — Supplementary Information [file 41467_2017_1144_MOESM1_ESM.pdf]

A) Targeting strategy

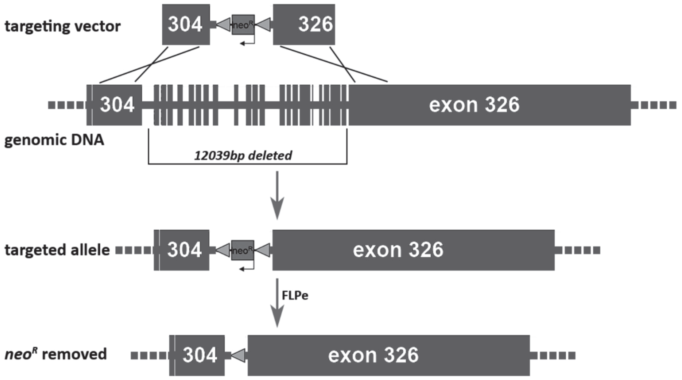

B) Genotypes of offspring--Het x Het breeding

Genotype	Male	Female	Total	Expected ratio
WT	37	25	**62**	1
Het	59	53	**112**	2
Ttn$^{\Delta C1\text{-}2}$	35	23	**58**	1
**Total**	**131**	**101**	**232**	
Deviates from Mendelian ratio?	No (p=0.5).	No (p=0.84).	No (p=0.81).	

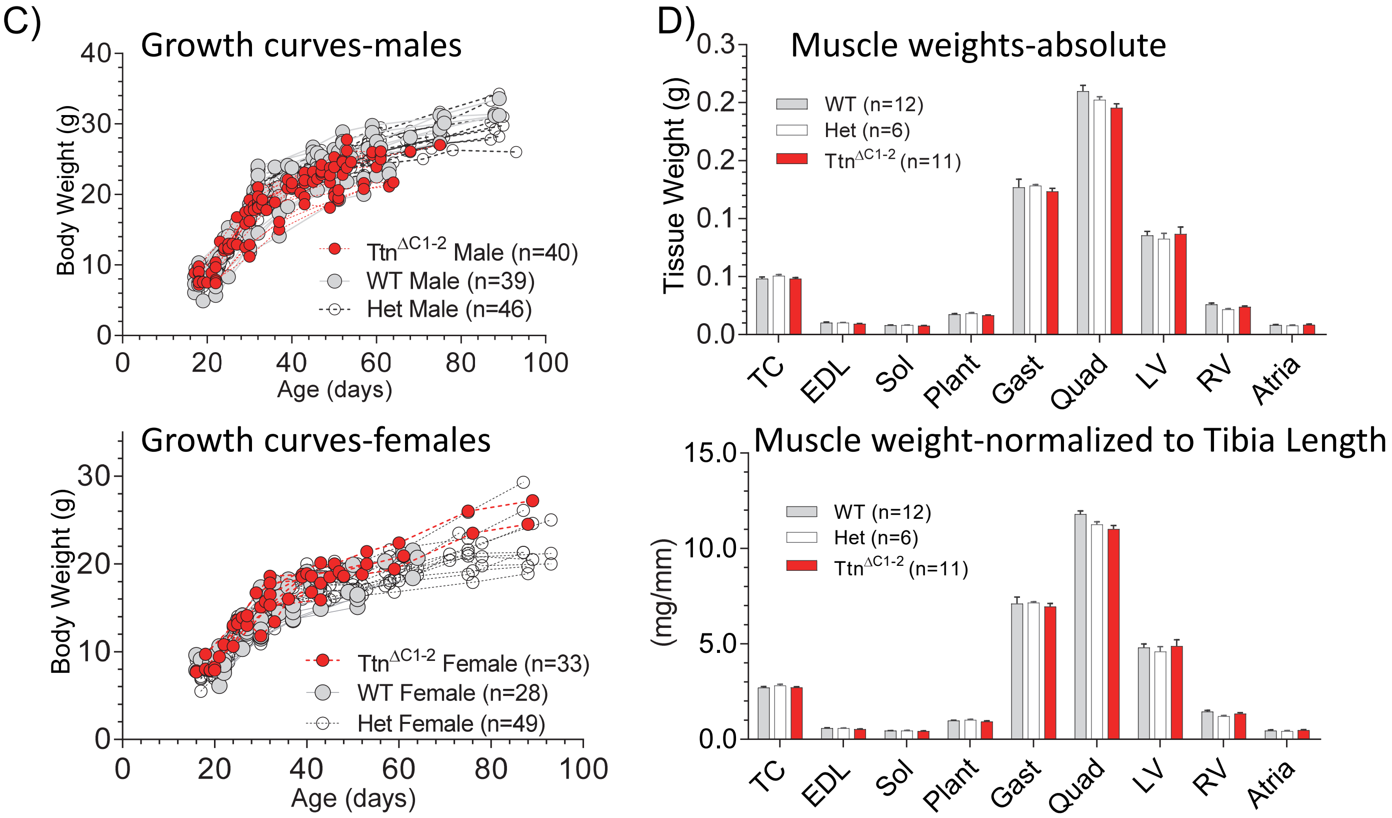

Supplementary Figure 1. Generation of the *Ttn$^{\Delta C1\text{-}2}$* mouse model. A) Targeting strategy used to delete exons 305-325 from the mouse gene (Ttn). B) Genotypes of offsprings of *Het×Het Ttn$^{\Delta C1\text{-}2}$* breeding are born at close to the expected Mendelian ratios (statistical comparison performed with chi-square test). C) Body weight growth curves of male (top) and female (bottom) *WT*, *Het* and *Ttn$^{\Delta C1\text{-}2}$* mice show no differences between genotypes (mice were used for *in vitro* studies at 8 weeks of age, explaining the drop-off in mouse numbers around that age). D) Muscle weight in 8 week old male mice with absolute weight (top) and Tibia Length normalized weights at the bottom. Error bars are s.e.m.

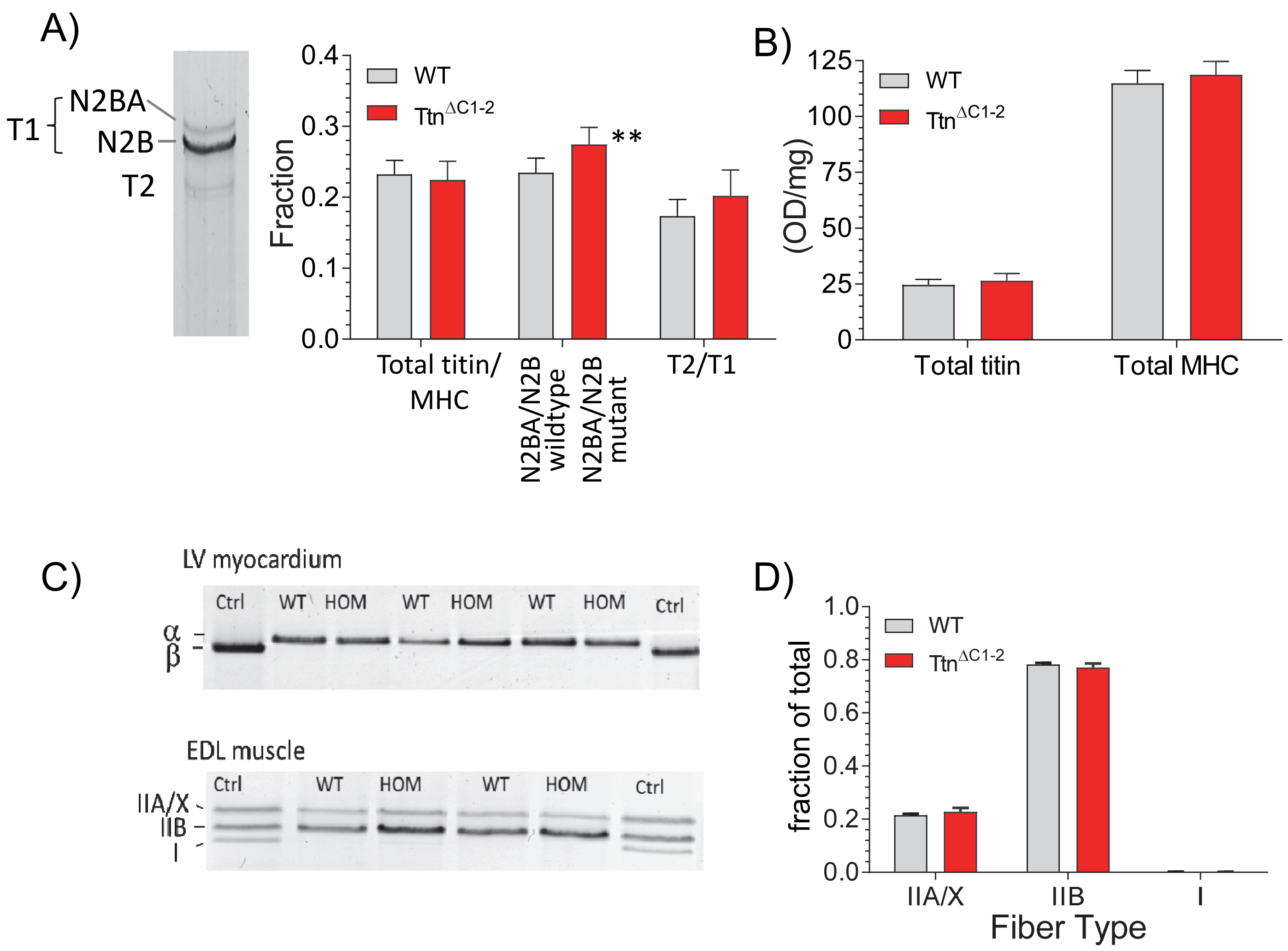

Supplementary Figure 2. Titin and MHC protein expression in cardiac and skeletal muscle from WT and homozygous *Ttn*$^{\Delta C1\text{-}2}$ mice. A and B) Titin expression in left ventricular myocardium (LV) of n=7 male and 3 female *WT*; n=7 male and 2 female *Ttn*$^{\Delta C1\text{-}2}$ 58-75 days old mice. A) Left shows a typical SDS-agarose gel revealing the two T1 titin isoforms N2B and N2BA titin as well as the T2 (assumed to be a titin degradation product). Right, comparison between *WT* and *Ttn*$^{\Delta C1\text{-}2}$ myocardium shows no differences in the total titin/myosin heavy chain (MHC) ratio, the N2BA/N2B ratio, or the T2/T1 (N2BA + N2B) ratio. B). The total titin per mg of solubilized tissue and the total amount of MHC are not different between *WT* and *Ttn*$^{\Delta C1\text{-}2}$ myocardium. C and D) myosin heavy chain isoform expression is visually not different in LV (top) nor EDL muscle (bottom). D) Quantitative analysis of MHC isoform expression in EDL muscle reveals no differences between genotypes. Ctrl in C is mixture of EDL and soleus lysate. 8 *WT* and 8 *Ttn*$^{\Delta C1\text{-}2}$ 58-75 days old mice (6 males and 2 females in each group) were used to study MHC protein expression. Mean values and ± s.e.m. are shown. Statistical analysis is performed with a two-tailed t-Test, details are provided in Table S4.

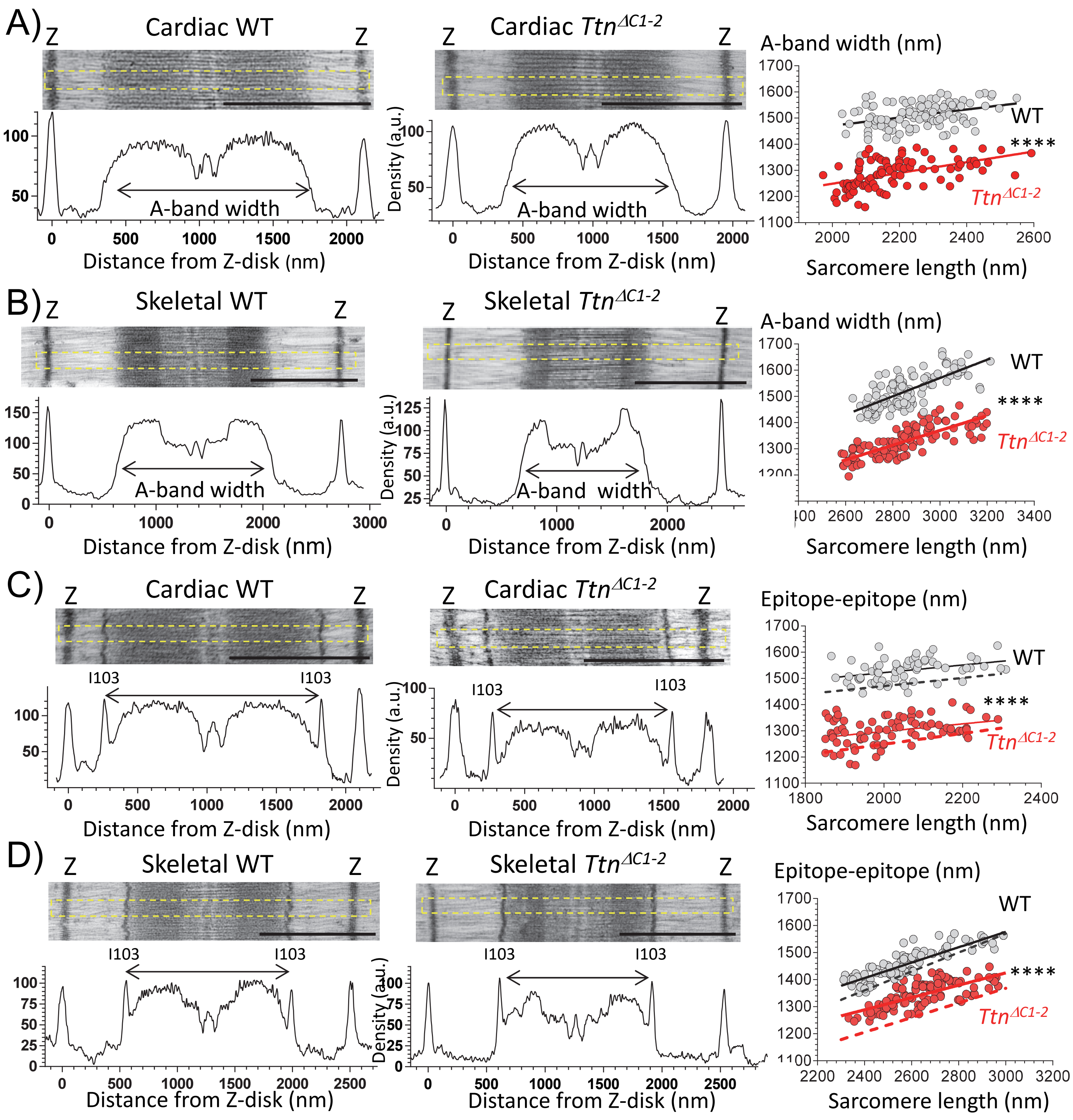

Supplementary Figure 3. A-band width measurements in LV myocardium and EDL muscle. A and B) Representative electron micrographs of cardiac and skeletal muscle sarcomere from *WT* and *Ttn*$^{\Delta C1-2}$ (top) with the profiles (bottom). Right, A-band width vs. sarcomere length data, fitted with linear regression lines. Offset of line fit of *Ttn*$^{\Delta C1-2}$ data is significantly less than that of *WT* ($p<0.0001$). C and D) Representative electron micrographs of *WT* and *Ttn*$^{\Delta C1-2}$ sarcomeres (top) labeled with I103 antibody. Density profile at bottom defines how epitope distances were measured. Right, Epitope distances vs sarcomere length data, fitted with linear regression lines. Offset of line fit of *Ttn*$^{\Delta C1-2}$ data is significantly less than that of *WT* ($p<0.0001$). The broken lines are the linear regression lines of the A and B panels. Statistical analysis is provided in Table S4. Scale bars: 1 µm. Male mice at 8 weeks of age (6 WT and 6 *Ttn*$^{\Delta C1-2}$) were used in this study.

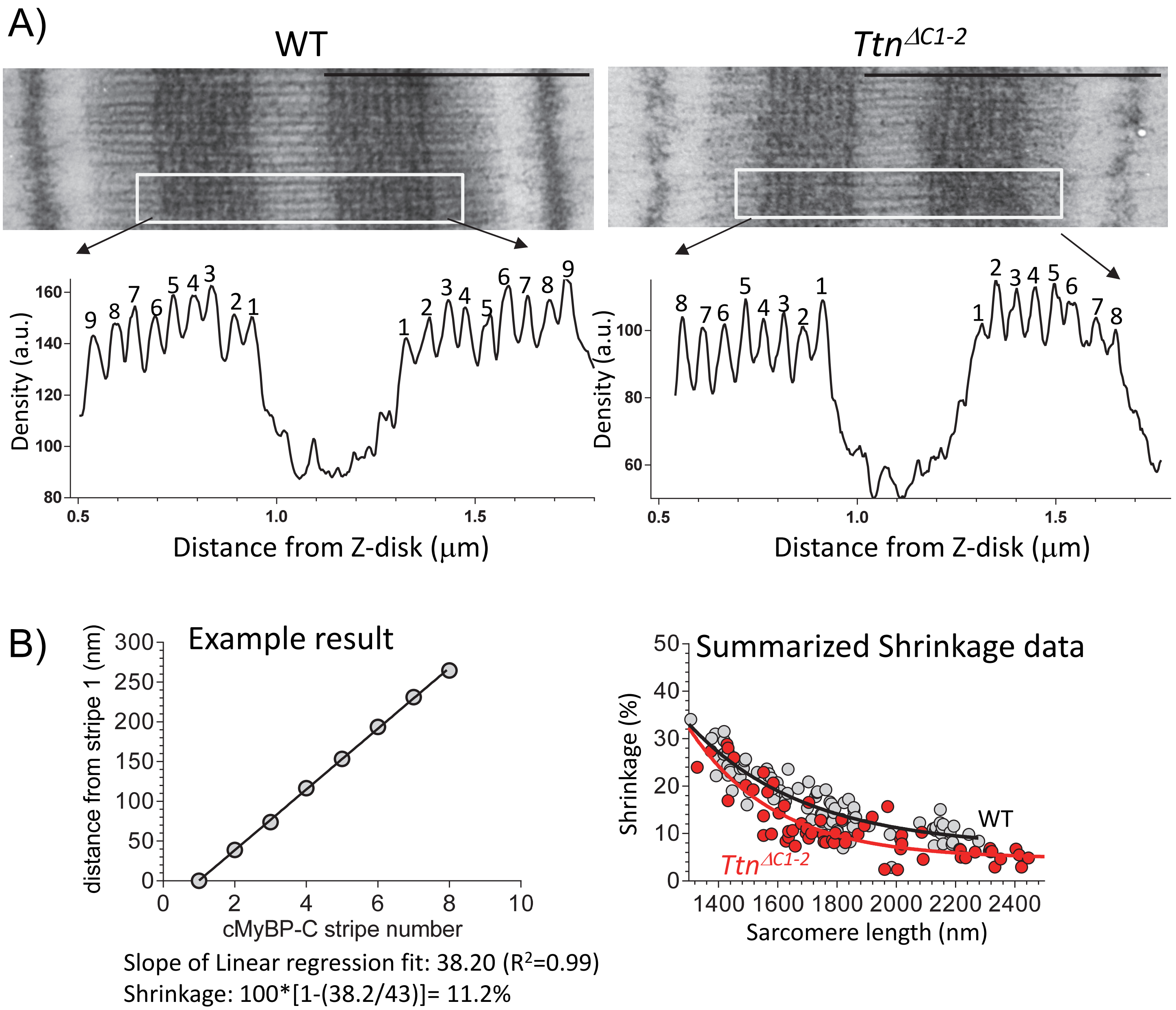

Supplementary Figure 4. A-band shrinkage correction in LV myocardium. A) Representative electron micrographs of sarcomeres labeled with anti cMyBP-C antibody revealing multiple stripes in the C-zone of the sarcomere. The spacing of these stripes was determined from the density profiles and assuming an *in vivo* spacing of 43 nm, measured A-band width data can be corrected for shrinkage. B) Left, Example result of A-band that was determined to have 11.2% shrinkage. Right, Summarized results show that shrinkage is large (~25 %) when sarcomere length is short (unstretched muscle) but in muscles that have been pre-stretched shrinkage is reduced to ~10%. Data were well fit with a one-phase decay curve (R^2 ~0.8). Comparison between *WT* and *Ttn*$^{\Delta C1\text{-}2}$ results showed that *Ttn*$^{\Delta C1\text{-}2}$ results had significantly less shrinkage ($p<0.0001$ using nonlinear curve comparison). Detailed statistical analysis is provided in Table S4. Scale bars: 1 μm. Male mice at 8 weeks of age (4 WT and 4 *Ttn*$^{\Delta C1\text{-}2}$) were used.

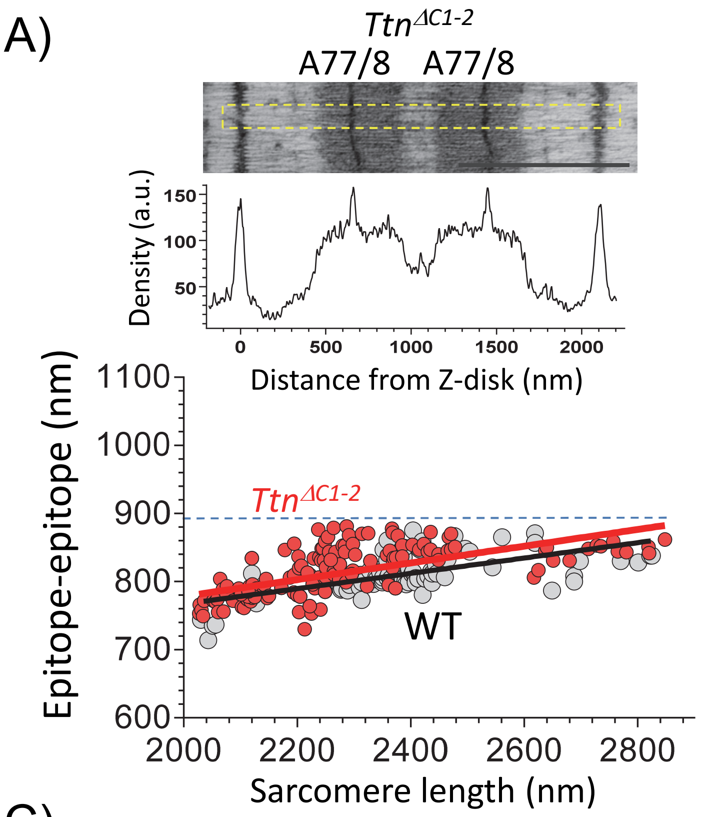

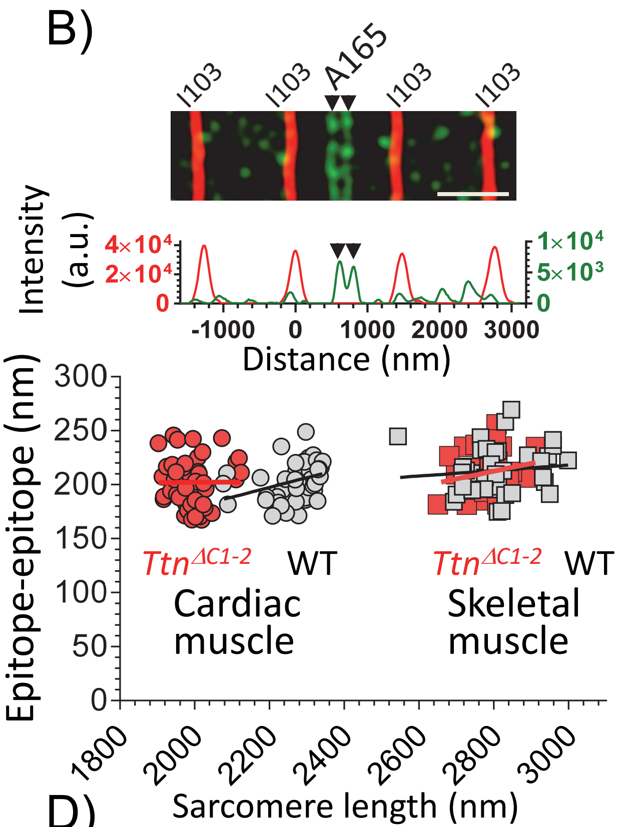

C)

A165 antibody

Cardiac (papillary)	WT	$Ttn^{\Delta C1-2}$	p-value
n	53	51	
Sarcomere Length (nm)	2274±8.6	1994±7.2	<0.0001
Epitope Distance (nm)	204.1±2.26	202.1±2.94	NS

Skeletal (EDL)	WT	$Ttn^{\Delta C1-2}$	p-value
n	39	36	
Sarcomere Length (nm)	2779±9.9	2811±14.8	NS
Epitope Distance (nm)	213.3±3.49	211.0±3.02	NS

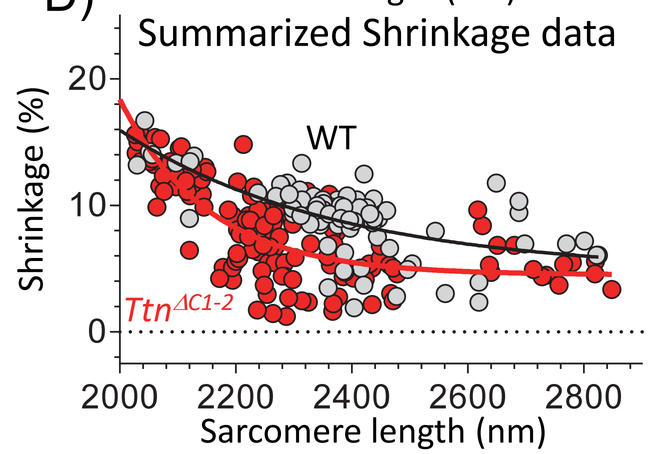

Supplementary Figure 5. A-band shrinkage correction in EDL skeletal muscle. A) Top, Representative electron micrograph labeled with anti A77/8 antibody revealing two epitopes in A-band region of the sarcomere. Bottom, Measured epitope distances versus sarcomere length. The broken horizontal line indicates the predicted *in vivo* distance between the epitopes (892 nm). B and C) Results obtained with the A165 antibody that labels the P-zone. Top of B) shows representative SIM image with the corresponding plot profile ($Ttn^{\Delta C1-2}$, EDL) and B) bottom summarized epitope-to-epitope distances for both cardiac muscle and EDL muscle; C) the summarized results show that in the EDL muscle the average distance is ~212 nm, two-tailed t-Test is used in statistics. The predicted *in vivo* A77/8 epitope-to-epitope distance was then determined as follows. The middle of the A77/8 fragment used to raise the antibody is 87 Ig/Fn domains from the middle of the A165 fragment and for the epitope to epitope distance this is 174 (87x2) domains. Considering that 11 domains span in vivo 43 nm, 174 domains span 680 nm (174/11*43). Because the A165 epitopes are ~212 nm apart across the M-band (as per Table C) the A77/8 epitope-to-epitope distance is predicted to be 892 nm (680 + 212 nm). D) Summarized A-band shrinkage data for EDL muscle. Data are well fit with a one-phase decay curve Y=(Y0-Plateau)*exp(-K*X)+Plateau. Comparison between *WT* and $Ttn^{\Delta C1-2}$ showed that $Ttn^{\Delta C1-2}$ results had significantly less shrinkage (p<0.0001 using nonlinear curve comparison). Mean values and ± s.e.m. are shown. Statistical analysis is provided in Table S4. Scale bars: 1 µm. 4 WT and 4 $Ttn^{\Delta C1-2}$ 60 days old male mice were used for both IEM and SIM studies.

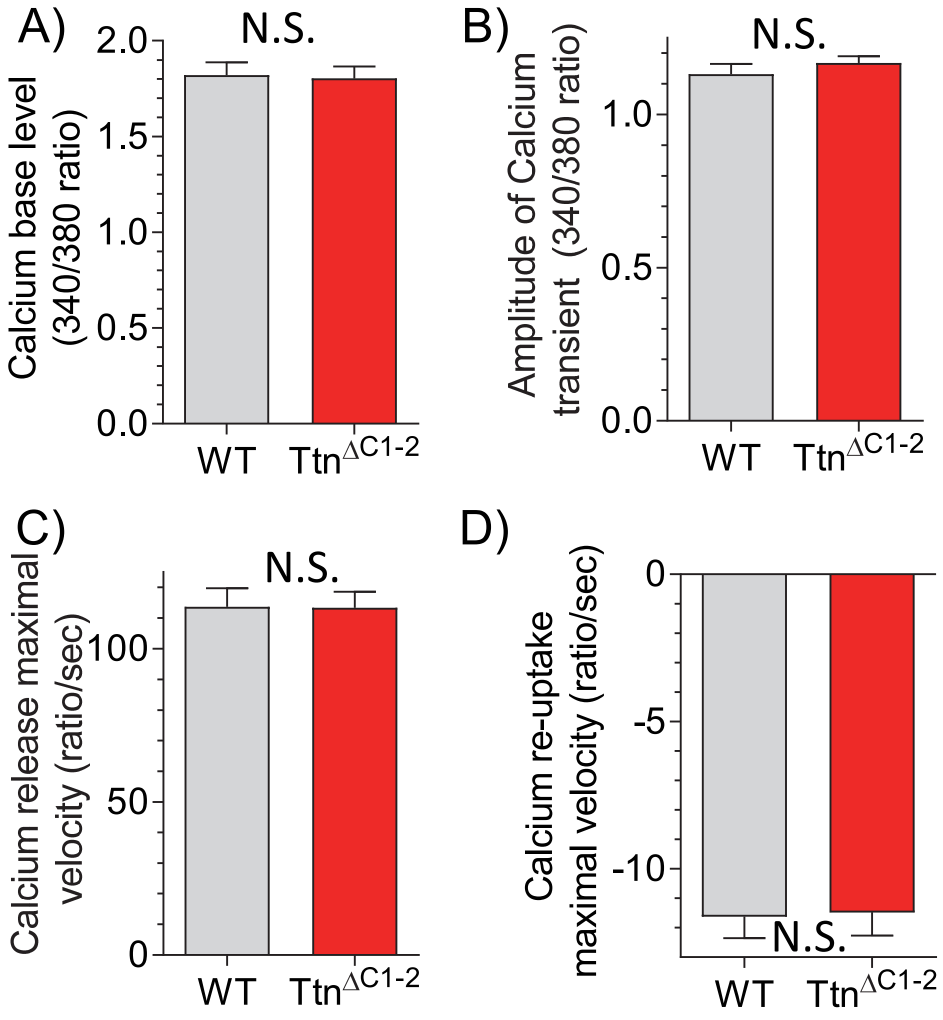

Supplementary Figure 6. Calcium transients. A - D) Fura-2 340/380 ratio of isolated intact cardiomyocytes stimulated at 2 Hz. A) Diastolic baseline signal level, B) Twitch amplitude, C) maximal velocity of rising, and D) maximal velocity of decaying signal are not different between *WT* and *Ttn*$^{\Delta C1-2}$ myocytes. N.S: not significant. Mean results from 5 hearts (20 cells per heart). Mean values and ± s.e.m. are shown. Statistical analysis with a two-tailed t-Test. 50 days old male mice were studied (n=5 *WT* and n=5 *Ttn*$^{\Delta C1-2}$).

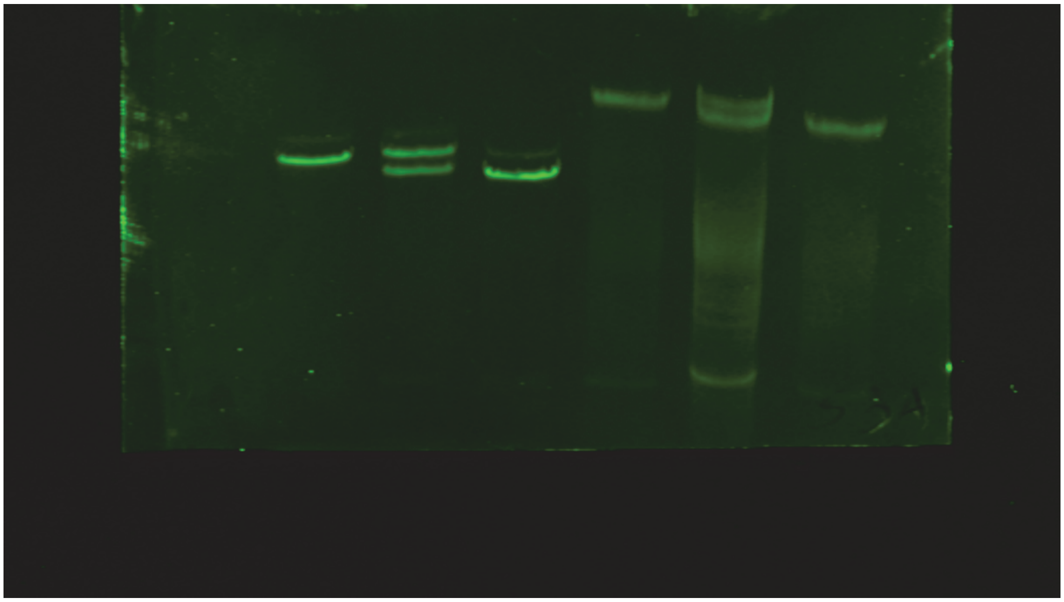

Supplementary Figure 7. Uncropped Western blot using Z1Z2 antibody.

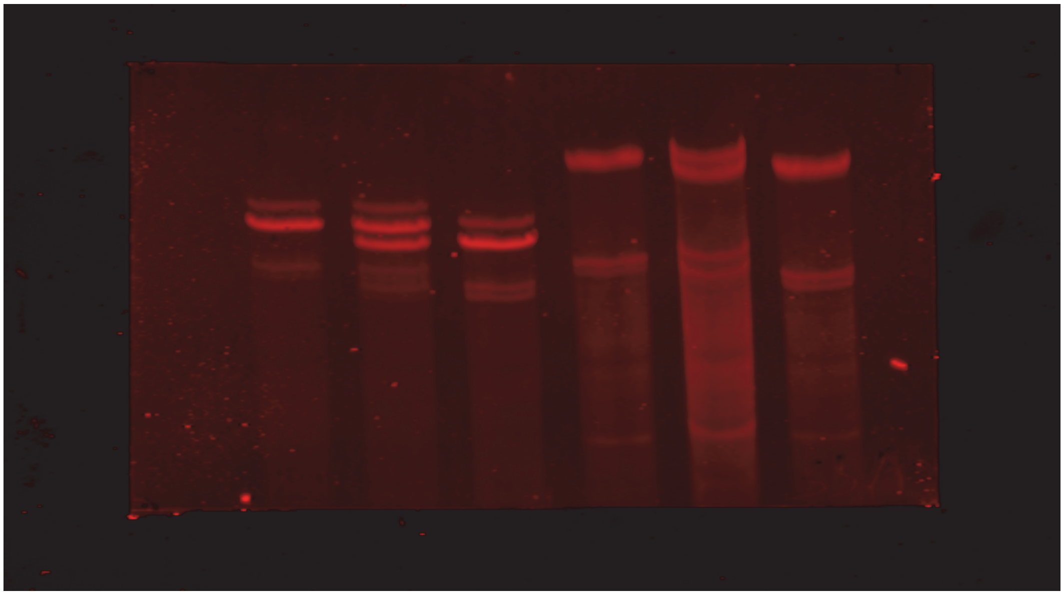

Supplementary Figure 8. Uncropped Western blot using M8-9 antibody.

	WT	*Ttn*$^{ΔC1-2}$	**p-value**
n	8	8	
Heart Rate (BPM)	533 ± 8	521 ± 11	0.536
Volume;s (µL)	25.6 ± 2.7	47.9 ± 5.3	0.004
Volume;d (µL)	71.7 ± 1.2	86.8 ± 5.0	0.022
Stroke Volume (µL)	46.1 ± 1.7	38.9 ± 0.9	0.006
Ejection Fraction (%)	64.6 ± 3.2	45.9 ± 2.8	0.002
Fractional Shortening (%)	35.3 ± 2.4	22.9 ± 1.6	0.002
Cardiac Output (mL/min)	24.5 ± 0.8	20.3 ± 0.5	0.001
LVAWd (mm)	0.91 ± 0.04	0.77 ± 0.03	0.039
LVAWs (mm)	1.47 ± 0.07	1.15 ± 0.05	0.005
LVIDd (mm)	3.99 ± 0.04	4.35 ± 0.09	0.006
LVIDs (mm)	2.50 ± 0.23	3.40 ± 0.15	0.014
LVPWd (mm)	0.80 ± 0.01	0.71 ± 0.03	0.018
LVPWs (mm)	1.31 ± 0.10	0.94 ± 0.05	0.009
LA (mm)	2.25 ± 0.06	2.16 ± 0.06	0.461
Eccentricity	4.7 ± 0.1	5.9 ± 0.3	0.002
A' (mm/s)	-29.4 ± 1.5	-29.8 ± 1.4	0.514
E' (mm/s)	-23.3 ± 1.1	-21.2 ± 1.9	0.624
A (mm/s)	589.2 ± 29.5	592.6 ± 17.1	0.596
MVdecel (ms)	25.8 ± 0.5	26.3 ± 0.6	0.219
E (mm/s)	763.0 ± 33.4	713.0 ± 26.3	0.428
E/A	1.30 ± 0.03	1.20 ± 0.03	0.029
E/E'	-32.8 ± 0.6	-35.0 ± 2.4	0.711

Supplementary Table 1. Echocardiographic analysis of the left ventricular chamber of the heart. s: systole; d: diastole; LV: left ventricle; AW: anterior wall; LVIDd: left ventricular internal diastolic diameter; PW: posterior wall; LA: left atrium; eccentricity: LVIDd/WTd; A’: Mitral annular late diastolic velocity; E’: Mitral annular early diastolic velocity; E: peak inflow velocity (from left atrium to left ventricle) of E-wave during early diastole; A: peak inflow velocity (from left atrium to left ventricle) of A-wave during late diastole; MVdecel: deceleration time of E-wave. Statistical analysis with a two-tailed t-Test. Mean values and ± s.e.m. are shown. Male *WT* (n=8) and *Ttn*$^{ΔC1-2}$ (n=8) mice at 50 days of age were studied.

Fura-2 340/380 ratio	***WT*** (n=5 mice, 103 cells)	***Ttn$^{\Delta C1-2}$*** (n=5 mice,111 cells)	**p value**
baseline	1.82±0.07	1.80±0.06	0.866
maximal departure velocity	113.7±6.1	113.4±5.2	0.974
time at max departure velocity (s)	0.005±0.000	0.006±0.000	0.401
peak	2.95±0.09	2.97±0.08	0.868
peak height	1.13±0.03	1.17±0.02	0.386
peak time (s)	0.019±0.001	0.020±0.001	0.494
maximal return velocity	-11.6±0.7	-11.5±0.8	0.901
time at max return velocity (s)	0.020±0.002	0.020±0.001	0.997
time to peak 50.0% (s)	0.006±0.000	0.006±0.000	0.312
time to peak 90.0% (s)	0.012±0.000	0.013±0.000	0.291
time to baseline 50.0% (s)	0.073±0.004	0.077±0.004	0.567
time to baseline 90.0% (s)	0.194±0.014	0.212±0.014	0.376
exponential decay time constant	0.119±0.004	0.119±0.006	0.948

Supplementary Table 2. The Fura-2 340/380 ratio of baseline diastolic calcium, amplitude and maximal velocity of calcium release or reuptake are not different. Mean values and ± s.e.m. are shown. Statistical analysis with a two-tailed t-Test. 50 days old male mice were studied (n=5 *WT* and n=5 *Ttn*$^{\Delta C1-2}$).

Antibody	Antigen	Source	Host and Clone
Z1Z2	Human titin domains Z1-2	Myomedix	Rabbit polyclonal
M8-9	Human titin domains M8-9	Myomedix	Rabbit polyclonal
Titin I103	Human titin domains I102-104	Gregorio (U of A)	Rabbit polyclonal
T12	Human titin domain I2	Boehringer	Mouse monoclonal
A77-8	Human titin domains A77-8	Granzier (U of A)	Rabbit polyclonal
A165	Human titin domain A165	Granzier (U of A)	Guinea Pig polyclonal
cMyBP-C	Human cMyBP-C domains C5-7	Harris (U of A)	Rabbit polyclonal
α-actinin	Human α-actinin (clone EA-53)	Sigma-Aldrich	Mouse monoclonal

Supplementary Table 3. Primary antibodies used in the present work.

Reference	n		Statistical Method	Value of the Test Statistic			p-value
	WT	***$Ttn^{\Delta C1-2}$***					
Fig. 2A; Cardiac m.	227	228	Linear Regression	Slope comparison (WT vs $Ttn^{\Delta C1-2}$)	F=0.71		p=0.40
				Intercept comparison (WT vs $Ttn^{\Delta C1-2}$)	F=4043		p<0.0001
Fig. 2A; Skeletal m.	273	269	Linear Regression	Slope comparison (WT vs $Ttn^{\Delta C1-2}$)	F=0.46		p=0.50
				Intercept comparison (WT vs $Ttn^{\Delta C1-2}$)	F=4514		p<0.0001
Fig. 2B; Cardiac	98	68	Two-tailed t-Test	t=39.40 (df=164)			p<0.0001
Fig. 2C; Skeletal	84	145	Two-tailed t-Test	t=13.47 (df=227)			p<0.0001
Fig. 3A	90	90	Nonlinear Regression	WT	R^2=0.94 (df=86)	One equation fits all?	p<0.0001
				$Ttn^{\Delta C1-2}$	R^2=0.93 (df=86)		
Fig. 3B	172	172	Segmental Linear Regression	WT	R^2=0.83 (df=170)	One equation fits all?	p<0.0001
				$Ttn^{\Delta C1-2}$	R^2=0.89 (df=170)		
Fig. 3C	101	100	Linear Regression	Slope: F=8.92			p=0.0092
Fig. 3E; ESP	7	8	Two-tailed t-Test	t=12.85 (df=13)			p<0.0001
Fig. 3E; ESPVR	7	8	Two-tailed t-Test	t=15.39 (df=13)			p<0.0001
Fig. 3E; PRSW	7	8	Two-tailed t-Test	t=23.77 (df=13)			p<0.0001
Fig. 3F; WTd	8	9	Two-tailed t-Test	t=9.63 (df=15)			p<0.0001
Fig. 3F; Eccentricity	8	9	Two-tailed t-Test	t=3.39 (df=15)			p=0.0041
Fig. 3F; EF	8	9	Two-tailed t-Test	t=12.75 (df=13)			p<0.0001
Table 1; Cardiac	98	68	Linear Regression	Slope	F=0.45		p=0.50
				Intercept	F=1566		p<0.0001
Table 1; Skeletal	84	145	Linear Regression	Slope	F=3.82		p=0.052
				Intercept	F=2109		p<0.0001
Supp. Fig. 2A	9	7	Two-tailed t-Test	TT/MHC	t=0.71 (df=14)		p=0.49
				N2BA/N2B	t=3.59 (df=14)		p=0.003
				T2/T1	t=1.91 (df=14)		p=0.08
Supp. Fig. 2B	9	7	Two-tailed t-Test	Total titin	t=1.32 (df=14)		p=0.21
				Total MHC	t=1.31 (df=14)		p=0.21
Supp. Fig. 2D	9	9	Two-tailed t-Test	IIA/X	t=2.24 (df=9.84)		p=0.05
				IIB	t=2.14 (df=10.36)		p=0.06
				I	t=0.71 (df=16)		p=0.49
Supp. Fig. 3A	108	93	Two-tailed t-Test	t=31.07 (df=199)			p<0.0001
Supp. Fig. 3B	118	115	Linear Regression	Slope	F=3.57		p=0.06
				Intercept	F=1340		p<0.0001
Supp. Fig. 3C	71	95	Two-tailed t-Test	t=30.86 (df=164)			p<0.0001
Supp. Fig. 3D	208	206	Two-tailed t-Test	t=21.58 (df=412)			p<0.0001
Supp. Fig. 4B	91	67	Nonlinear Regression	WT	R^2=0.82 (df=88)	One equation fits all?	p<0.0001
				$Ttn^{\Delta C1-2}$	R^2=0.74 (df=64)		
Supp. Fig. 5A	93	161	Linear Regression	Slope	F=0.24		p=0.63
				Intercept	F=16.6		p<0.0001
Supp. Fig. 5B; Cardiac	53	51	Linear Regression	Slope	F=1.77		p=0.19
				Intercept	F=1.93		p=0.17
Supp. Fig. 5B; Skeletal	39	36	Linear Regression	Slope	F=0.48		p=0.49
				Intercept	F=0.05		p=0.83
Supp. Fig. 5C; Cardiac	53	51	Two-tailed t-Test	SL	t=24.94 (df=102)		p<0.0001
				Ep. dist.	t=0.55 (df=102)		p=0.58
Supp. Fig. 5C; EDL	36	39	Two-tailed t-Test	SL	t=1.8 (df=65.44)		p=0.076
				Ep. dist.	t=0.49 (df=73)		p=0.63
Supp. Fig. 5D	90	139	Nonlinear Regression	WT	R^2=0.41 (df=87)	One equation fits all?	p<0.0001
				$Ttn^{\Delta C1-2}$	R^2=0.62 (df=136)		

Supplementary Table 4. Statistical analysis. Symbols on Figures **: p<0.01; ***:p<0.001; ****: p<0.0001.
